# A climate-sensitive, contemporary multi-sphere bacterial metacommunity across the Tibetan Plateau

**DOI:** 10.1093/nsr/nwag463

**Published:** 2026-08-03

**Authors:** Yongqin Liu, Mengdie Geng, Peng Xing, Yunfeng Yang, Mukan Ji, Pengfei Liu, Keshao Liu, Jin Zeng, Jianjun Wang, Yong-Guan Zhu, Tandong Yao

**Affiliations:** Center for Pan-third Pole Environment, Lanzhou University, Lanzhou 730000, China; State Key Laboratory of Tibetan Plateau Earth System, Environment and Resources (TPESER), Institute of Tibetan Plateau Research, Chinese Academy of Sciences, Beijing 100101, China; Chayu Integrated Observation and Research Station of the Xizang Autonomous Region, Linzhi 860600, China; Key Laboratory of Pan-third Pole Biogeochemical Cycling, Lanzhou 730000, China; Center for Pan-third Pole Environment, Lanzhou University, Lanzhou 730000, China; Chayu Integrated Observation and Research Station of the Xizang Autonomous Region, Linzhi 860600, China; Key Laboratory of Pan-third Pole Biogeochemical Cycling, Lanzhou 730000, China; Key Laboratory of Lake and Watershed Science for Water Security, Nanjing Institute of Geography and Limnology, Chinese Academy of Sciences, Nanjing 211135, China; Tsinghua Shenzhen International Graduate School, Shenzhen 518055, China; Center for Pan-third Pole Environment, Lanzhou University, Lanzhou 730000, China; State Key Laboratory of Tibetan Plateau Earth System, Environment and Resources (TPESER), Institute of Tibetan Plateau Research, Chinese Academy of Sciences, Beijing 100101, China; Chayu Integrated Observation and Research Station of the Xizang Autonomous Region, Linzhi 860600, China; Key Laboratory of Pan-third Pole Biogeochemical Cycling, Lanzhou 730000, China; Center for Pan-third Pole Environment, Lanzhou University, Lanzhou 730000, China; State Key Laboratory of Tibetan Plateau Earth System, Environment and Resources (TPESER), Institute of Tibetan Plateau Research, Chinese Academy of Sciences, Beijing 100101, China; Chayu Integrated Observation and Research Station of the Xizang Autonomous Region, Linzhi 860600, China; Key Laboratory of Pan-third Pole Biogeochemical Cycling, Lanzhou 730000, China; State Key Laboratory of Tibetan Plateau Earth System, Environment and Resources (TPESER), Institute of Tibetan Plateau Research, Chinese Academy of Sciences, Beijing 100101, China; Key Laboratory of Lake and Watershed Science for Water Security, Nanjing Institute of Geography and Limnology, Chinese Academy of Sciences, Nanjing 211135, China; Key Laboratory of Lake and Watershed Science for Water Security, Nanjing Institute of Geography and Limnology, Chinese Academy of Sciences, Nanjing 211135, China; Research Center for Eco-Environmental Sciences, Chinese Academy of Sciences, Beijing 100085, China; Center for Pan-third Pole Environment, Lanzhou University, Lanzhou 730000, China; State Key Laboratory of Tibetan Plateau Earth System, Environment and Resources (TPESER), Institute of Tibetan Plateau Research, Chinese Academy of Sciences, Beijing 100101, China

**Keywords:** Tibetan Plateau, multi-sphere, microbes, biodiversity, annual mean precipitation

## Abstract

The Tibetan Plateau hosts interacting atmospheric, glacier, river, lake, and soil ecosystems, yet the organization and climatic sensitivity of their bacterial communities remain unclear. Here, we compiled a 16S rRNA gene dataset of 3373 samples across five ecosystems and nine habitats. More than 55% of Operational Taxonomic Units occurred in at least two ecosystems, and ∼6% in all five, thus revealing a metacommunity in which aerosols seed glacier microbiomes, and glaciers act as sinks, filters, and relays, exporting microbes to rivers, lakes, and soils. Nevertheless, community uniqueness and core microbiomes demonstrate robust habitat filtering and distinct biogeographic fingerprints. Mean annual precipitation exerts more potent effects on richness and community uniqueness than temperature or solar radiation, but exerts opposite influences on bacterial beta diversity in glacier versus downstream ecosystems. These findings provide novel insights into microbial interconnectedness and climate sensitivity in the Tibetan Plateau.

## INTRODUCTION

The Tibetan Plateau is the youngest and highest plateau on Earth, covering ∼3.08 × 10^6^ km^2^ with an average elevation of about 4400 m [1]. It contains glaciers, permafrost, wetlands, lakes, and rivers, forming one of the most extensive mid- and low-latitude cryosphere–hydrosphere complexes and a natural laboratory for cross-ecosystem research [2]. The Tibetan Plateau, known as the ‘Asian water tower’, hosts the largest mid- and low-latitude glacier and permafrost complexes, with modern glaciers covering about 49 873 km^2^ and permafrost extending over roughly 1.3 million km^2^ [2,3]. In addition, 1171 lakes larger than 1 km^2^ constitute a key component of this water tower, together covering ∼46 527 km^2^, or about 1.8% of the plateau’s surface [4,5]. These cryospheric and hydrological components are tightly coupled through exchanges of matter and energy among the atmosphere, glaciers, permafrost, rivers, and lakes, forming an atmosphere–cryosphere–land–water continuum that regulates both the quantity and quality of water exported from the plateau [6]. The Tibetan Plateau is also one of the most sensitive regions on Earth to climate change, with temperatures increasing at twice the global average [7]. Understanding how this multi-sphere continuum is organized and how it responds to environmental forcing is therefore critical for assessing the stability of regional water resources, biodiversity, and carbon cycling [8].

Microorganisms are widely recognized as effective biological proxies for studying ecosystem responses to climate change [9,10] because they can persist and remain metabolically active across a broad range of extreme environments [11]. On the Tibetan Plateau, microbial communities inhabit all major spheres—air, ice, water, and soils—and mediate key processes in carbon and nutrient cycling. Existing studies, however, have mostly focused on individual ecosystems in isolation, including glaciers [12], lakes [13], rivers [14], permafrost [15], and airborne communities [16]. While these studies have identified dominant and rare microbial taxa within a single or a few habitats, they provide only a fragmented view of the plateau’s microbiome. We still lack an integrated assessment of how bacterial communities are organized along the atmosphere–glacier–downstream continuum, how strongly spheres are connected by dispersal, and how habitat filtering and core microbiomes imprint distinct biogeographic signatures on this connectivity. Such a multi-sphere perspective is particularly important for understanding how cryosphere retreat and hydrological reorganization propagate microbiome change from glaciers to downstream rivers, lakes, and soils [17,18].

With a warming rate twice the global average and second only to the Arctic [19], the Tibetan Plateau has undergone pronounced environmental change, including rapid glacier retreat, permafrost thaw [20], and markedly altered land-atmosphere interactions [21]. As the invisible majority, microorganisms respond to ecosystem dynamics and human activities [22]. Significant changes in the plateau’s ecosystems exert a range of impacts on the local and regional distributions of microbes [23–25], while the loss of microbial lineages adapted to extreme environments may have far-reaching consequences for ecosystem functions [26]. Yet, it remains unclear how large-scale climatic gradients—particularly precipitation, which governs water availability, atmospheric deposition, and hydrological connectivity—shape the diversity, community uniqueness, and spatial turnover of bacterial communities across spheres. It is likewise unresolved whether the plateau’s microbiome behaves as a connected, climate-sensitive metacommunity or as loosely linked, locally assembled communities [27,28].

Here, we compile a communal catalog to characterize bacterial diversity and potential cross-ecosystem associations across the Tibetan Plateau. We integrate 16S rRNA gene amplicon sequencing data from 3373 samples spanning five representative ecosystems (Fig. 1): the atmosphere, glaciers, rivers, lakes, and soils. This facilitates comparative analyses of the microbial community composition, biodiversity, and community stability across nine distinct habitats: ice-snow, cryoconite, glacial runoff, aerosol, soil, river water, river sediment, lake water, and lake sediment. By integrating multi-habitat biodiversity data with climatic gradients, we provide the first plateau-wide perspective on the Tibetan Plateau as a multi-sphere bacterial metacommunity. It offers a process-based entry point for exploring how microbial connectivity, habitat filtering, and climate interact to shape the ecosystem stability and functioning of the ‘Asian water tower’.

**Figure 1.**
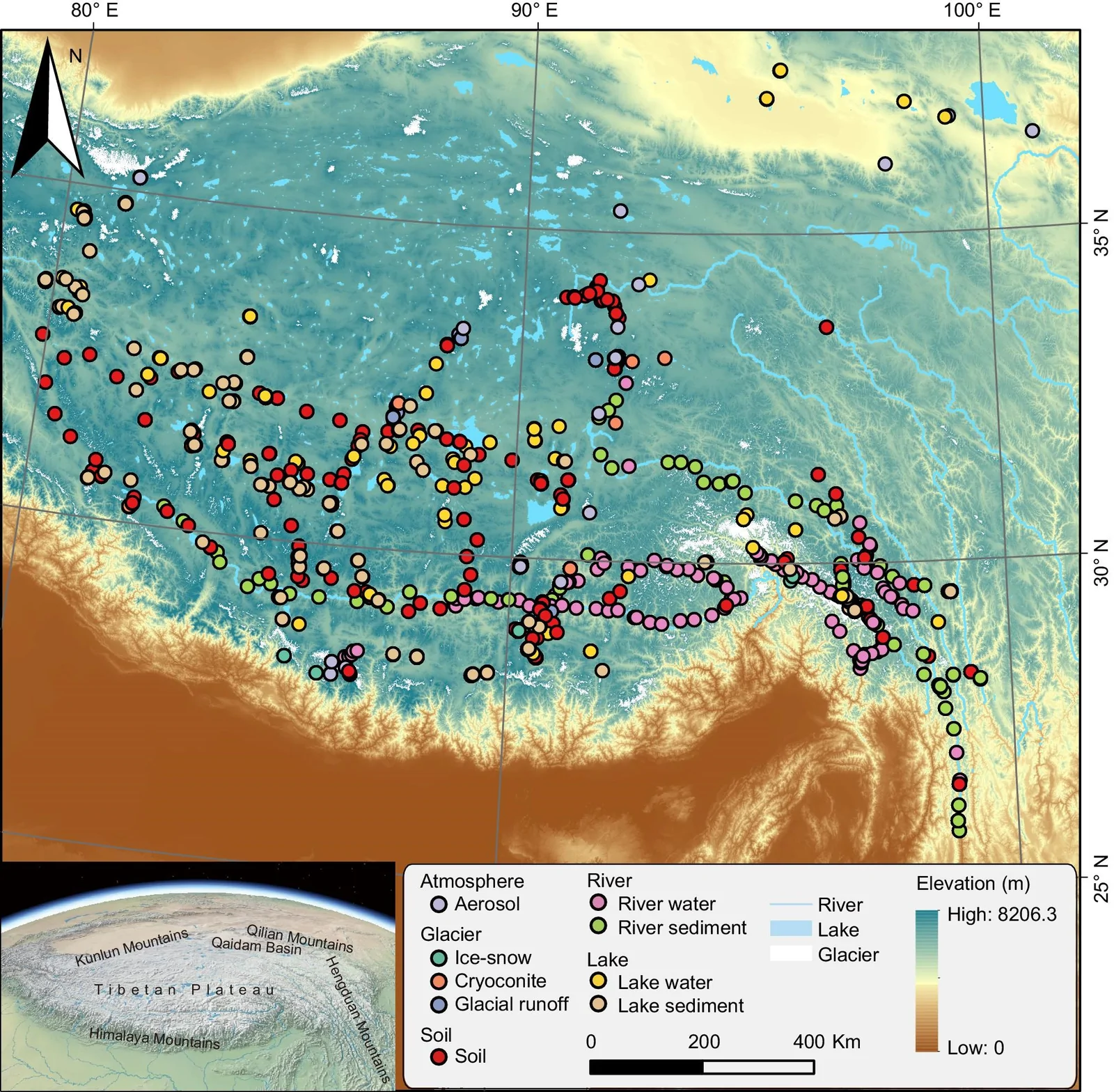
The geographical scope of the Tibetan Plateau and the spatial distribution of samples (*n* = 3373) across the five representative ecosystems (atmosphere, glacier, river, lake, and soil) and nine distinct habitats (aerosol, ice-snow, cryoconite, glacial runoff, river water, river sediment, lake water, lake sediment, and soil) on the Tibetan Plateau. The map was created using ArcGIS Pro 3.0.2.

## RESULTS

### Connectivity of bacterial communities among five ecosystems

Across the Tibetan Plateau, bacterial communities showed extensive overlaps among atmosphere, glaciers, lakes, rivers, and soils, indicating strong potential connectivity along the atmosphere–glacier–downstream continuum (Fig. 2). After quality control, 97 383 632 high-quality reads were retained and clustered at a 97% similarity threshold into 795 988 bacterial Operational Taxonomic Units (OTUs). Of these, 56.9% were detected in at least two ecosystems (Fig. 2a and Fig. S1). The atmosphere, glaciers, lakes, rivers, and soils accounted for 16.7%, 36.3%, 49.4%, 53.2%, and 55.6% of the total OTUs, respectively. In contrast, OTUs unique to these ecosystems accounted for 1.9%, 6.6%, 9.8%, 9.2%, and 15.6% of the total OTUs, respectively (Fig. 2a). A total of 46 305 OTUs were found across all five ecosystems, representing 5.8% of the total OTUs.

**Figure 2.**
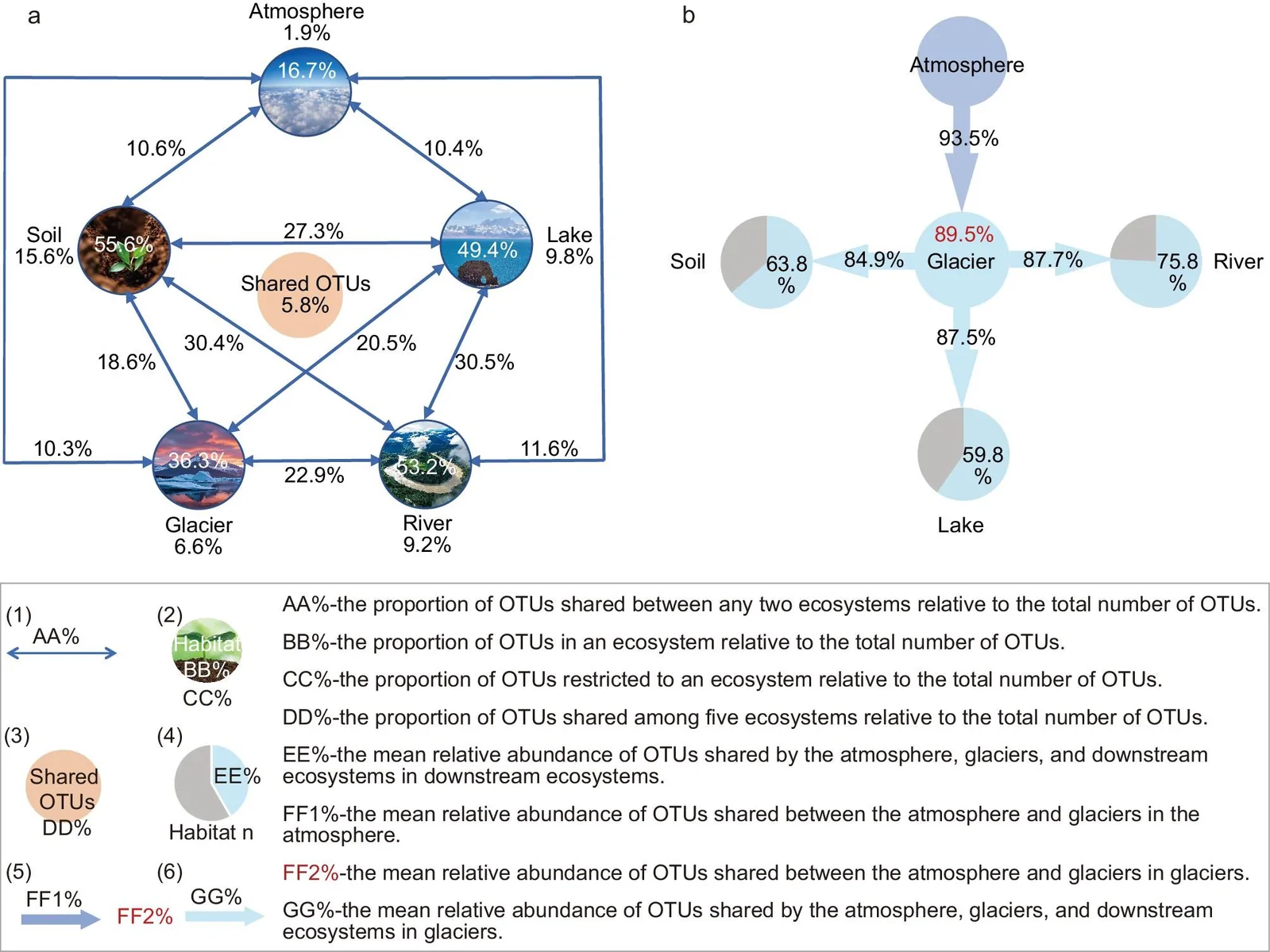
Multi-sphere connections among five ecosystems. (a) Overlap of OTUs across five ecosystems. Numbers on connecting lines represent the percentage of OTUs shared between pairwise ecosystems relative to the total number of OTUs. Shared OTUs were those occurring in all five ecosystems. (b) Associations between glaciers and the other ecosystems. Arrows indicate the hypothetical direction of OTU transport. The right section of each soil, river, and lake pie chart represents the relative abundance of OTUs shared by both the atmosphere and glaciers within that habitat.

Within each ecosystem, OTU overlap among habitats was also substantial (Fig. S2). In the glacier ecosystem, ∼40% of the glacial OTUs were shared among at least two habitats, and 14.7% occurred in all three (Fig. S2a). 25.4%, 18.9%, and 17.8% of the glacial OTUs were unique to cryoconite, ice-snow, and glacial runoff, respectively. In river ecosystems, 36.5% of river OTUs were shared across habitats, whereas 32.5% and 31.0% were unique to river water and sediment, respectively (Fig. S2b). In lake ecosystems, 24.5% of lake OTUs were shared between habitats, with 50.1% and 25.4% unique to sediment and water, respectively (Fig. S2c).

At the cross-ecosystem scale, 10.3% of all OTUs were shared between the glaciers and atmosphere (Fig. 2a), which accounted for 93.5% and 89.5% of the total OTU abundance detected in the atmosphere and glaciers, respectively (Fig. 2b). As shown in the conceptual model with the glacier ecosystem serving as the intermediate system, OTUs shared by the glaciers and atmosphere and also detected in the soils exhibited a mean relative abundance of 84.9% in the glaciers and 63.8% in the soils (Fig. 2b). Similarly, OTUs shared by the glaciers and atmosphere and also occurring in the lakes showed a mean relative abundance of 87.5% in the glaciers and 59.8% in the lakes. OTUs shared among the glaciers, atmosphere, and rivers accounted for a mean relative abundance of 87.7% in the glaciers and 75.8% in the rivers. Overall, these shared OTUs consistently accounted for a high proportion of the microbial communities in the glacier ecosystem and remained abundant in the downstream ecosystems, particularly in rivers. Alternative models in which the soil, lake, and river serve as intermediate systems are presented in Supplementary Results R1 and in Figs S3 and S4. Vertical comparisons indicate a tighter microbial connection from the atmosphere to glaciers and onward to downstream aquatic and terrestrial ecosystems on the plateau (Fig. 2b).

### Bacterial community composition on the Tibetan Plateau

Across all samples, the 795 988 OTUs were assigned to 85 bacterial phyla. The most abundant phylum was Proteobacteria (37.6%), Bacteroidota (16.5%), and Actinobacteriota (13.4%), followed by Cyanobacteria (5.7%), Firmicutes (5.5%), Acidobacteriota (4.9%), Chloroflexi (3.9%), and Planctomycetota (2.9%) (Fig. 3). At the order level, Burkholderiales was the most abundant (15.1%), followed by Flavobacteriales (6.6%) and Sphingomonadales (5.6%), Pseudomonadales (4.7%), and Micrococcales (4.2%) (Fig. S5). At the genus level, *Flavobacterium* was the most abundant (5.1%), followed by *Polaromonas* (2.8%), *Rhodoferax* (2.4%), *Acinetobacter* (2.3%), *Sphingorhabdus* (2.1%), *Sphingomonas* (1.9%), *Cyanobium PCC-6307* (1.7%), *hgcI clade* (family Sporichthyaceae, 1.3%), *Brevundimonas* (1.3%), *Limnohabitans* (1.1%), *CL500-29 marine group* (family Ilumatobacteraceae, 1.1%), *Massilia* (1.1%), and *Pseudomonas* (1.0%) (Fig. S6). Additional details on the distribution of the dominant orders and genera are presented in Supplementary Results R2 and R3. The relative abundance of the top 8 most abundant phyla (relative abundance >2%) varied markedly across the nine habitats (Supplementary Results R4 and Fig. S7).

**Figure 3.**
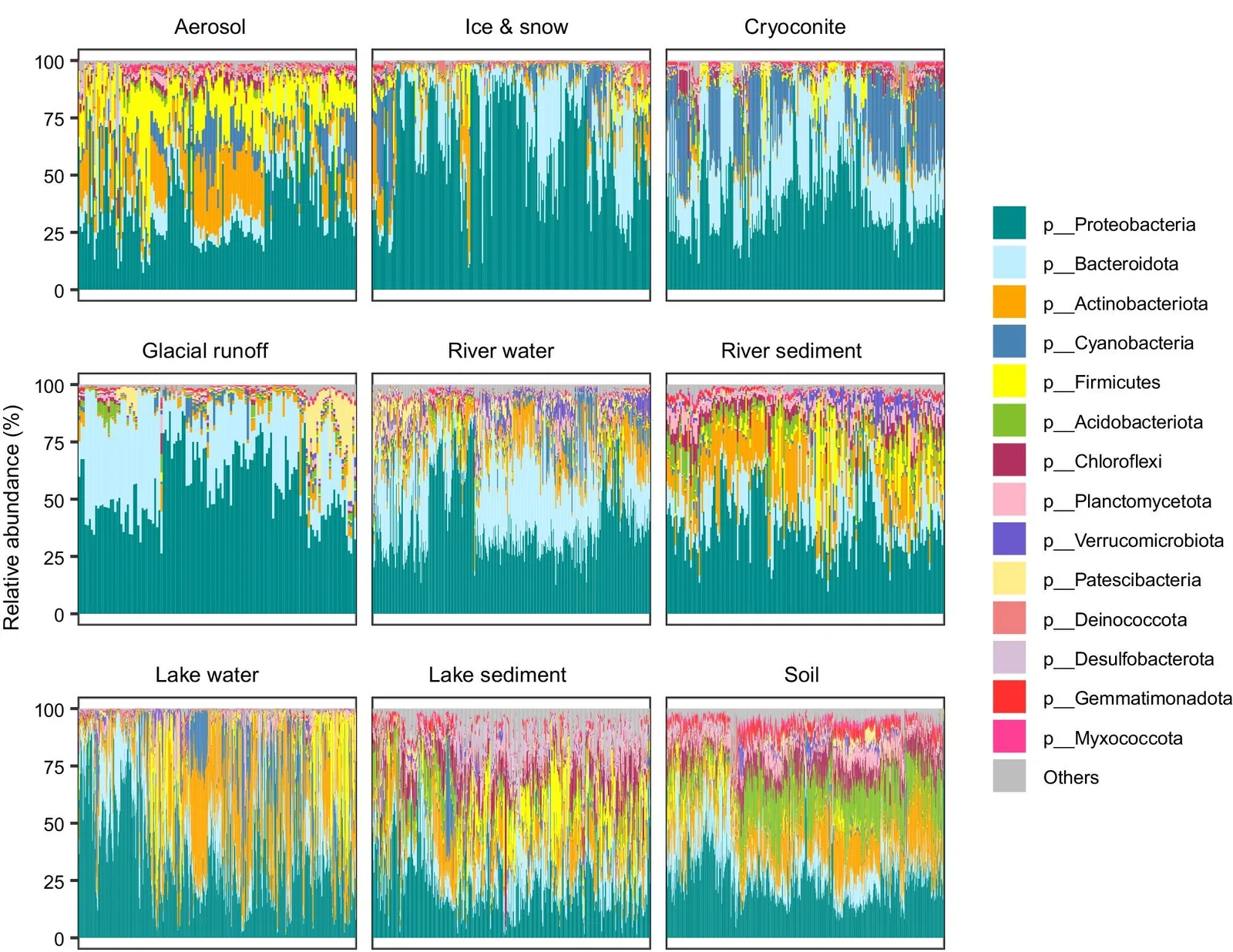
Relative abundances of the most abundant bacterial phyla across the nine habitat types.

### Bacterial diversity in each habitat

Bacterial richness varied significantly across nine habitats (Fig. 4a). River sediment and soil had the highest richness (2101 ± 620 and 2080 ± 740, respectively), followed by river water (1742 ± 579), glacial runoff (1580 ± 578), lake sediment (1449 ± 638), cryoconite (1347 ± 280), and aerosol (1186 ± 628). In contrast, ice-snow and lake water had the lowest richness (968 ± 379 and 735 ± 420, respectively). Other alpha diversity indices, such as community evenness, the Shannon–Wiener index, and the Simpson index, closely mirrored richness across habitats (Fig. S8). To systematically assess gamma diversity within habitats, we used asymptotic estimates based on incidence data. On average, we sampled 64.4% (interquartile range 58.1%–66.2%) of the estimated OTUs within each habitat, indicating that our sampling effort is broadly representative and allows robust comparisons of bacterial biodiversity across habitats (Fig. 4b).

**Figure 4.**
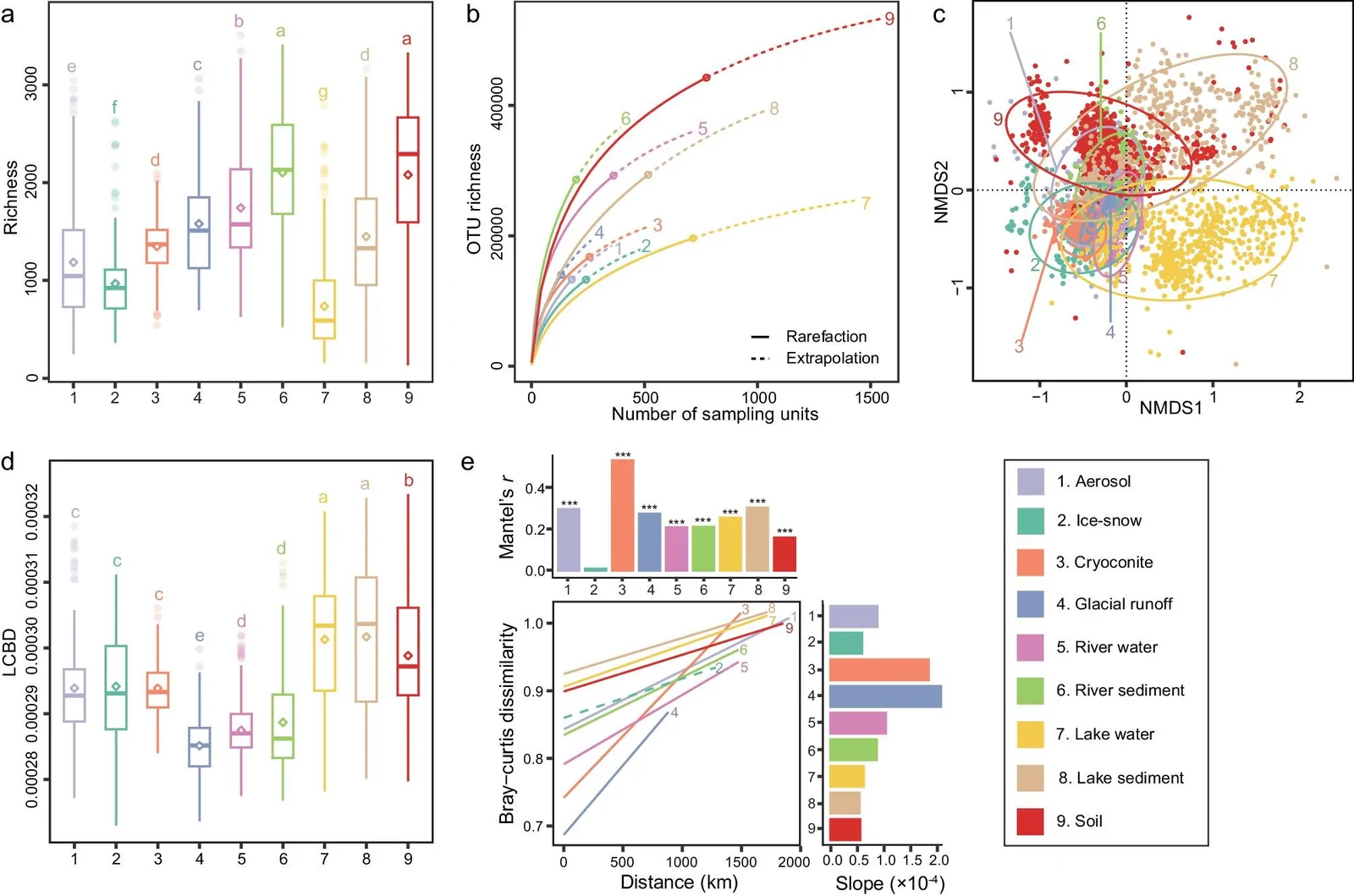
Bacterial diversity, community composition, and spatial distance–decay relationships across nine habitat types. (a) Differences in the bacterial richness among the nine habitat types. Different letters indicate significant differences at the *P* < 0.05 level determined by nonparametric Wilcoxon tests. Boxplots show the 25th and 75th percentiles (box limits), median (line), and mean (white dot). (b) Observed and estimated gamma diversity based on asymptotic estimates for each habitat. (c) Nonmetric multidimensional scaling (NMDS) based on Bray–Curtis dissimilarity matrices reveals differences in community composition among the nine habitat types. (d) Differences in LCBD among the nine habitat types. Different letters indicate significant pairwise differences at the *P* < 0.05 level determined by nonparametric Wilcoxon tests. The boxplot components are as in (a). (e) Spatial distance–decay relationships for nine habitat types. Statistically significant relationships by Mantel tests are shown with solid lines (*P* < 0.05), while dashed lines show non-significant relationships (*P* ≥ 0.05). The top panel displays the values of Mantel's *r* for the spatial distance–decay relationships, and the right panel shows the slopes of the ordinary least-squares regression lines fitted to the relationship between spatial distance and community dissimilarity.

Habitat-specific differences in community composition were evident based on non-metric multidimensional scaling (NMDS) and permutational analysis of variance (PERMANOVA) (Fig. 4c and Table S3; *P* < 0.05). Local contributions to beta diversity (LCBD) further revealed differences in community uniqueness among habitats (Fig. 4d; *P* < 0.05). Lake sediment, lake water, and soil contributed the most to beta diversity, followed by aerosol, ice-snow, river sediment, river water, cryoconite, and glacial runoff. LCBD was generally negatively associated with richness across habitats, with a slightly curvilinear pattern (Fig. S9). Notably, lake water exhibited significantly lower richness with marked community uniqueness, suggesting that species-poor sites tend to contribute the most to the local-to-regional beta diversity. To address potential biases arising from differences in sample sizes across habitats, we repeated analyses using a resampling procedure (*n* = 100 per habitat, Supplementary Methods M3.3). We found that richness and community uniqueness were generally consistent with those in the full dataset (Fig. S10).

Across spatial scales ranging from local (0 m) to over 2000 km, eight of nine habitats, excluding ice-snow, exhibited significant distance decay in community similarity. The intensities of distance–decay, quantified by the *r* values of the Mantel test, were highest (0.530) in cryoconite, followed by lake sediment (0.301), aerosol (0.294), glacial runoff (0.272), lake water (0.252), river sediment (0.208), river water (0.206), and soil (0.156) (Fig. 4e).

### Core microbial taxa in nine habitats

We defined core OTUs within each habitat as the top 10% most abundant OTUs that occurred in more than 50% of the samples (Delgado-Baquerizo *et al.* 2018). A total of 1757 core OTUs were identified across nine habitats, of which 1123 (63.9%) were exclusive to a single habitat (Fig. 5a and Fig. S12). A total of 352 core OTUs (20.0%) were present in two habitats, and 282 core OTUs (16.1%) were present in at least three habitats. Only four core OTUs were detected across all nine habitats and taxonomically affiliated with *Polaromonas, Cryobacterium, Rhodoferas*, and *Pseudarthrobacter* (Table S2). In addition, the taxonomic affiliations of core OTUs varied considerably among habitats (Fig. 5a and Fig. S12). Additional details on the phylogenetic relationships of core OTUs between and among habitats are provided in the Supplementary Results R6 and R7.

**Figure 5.**
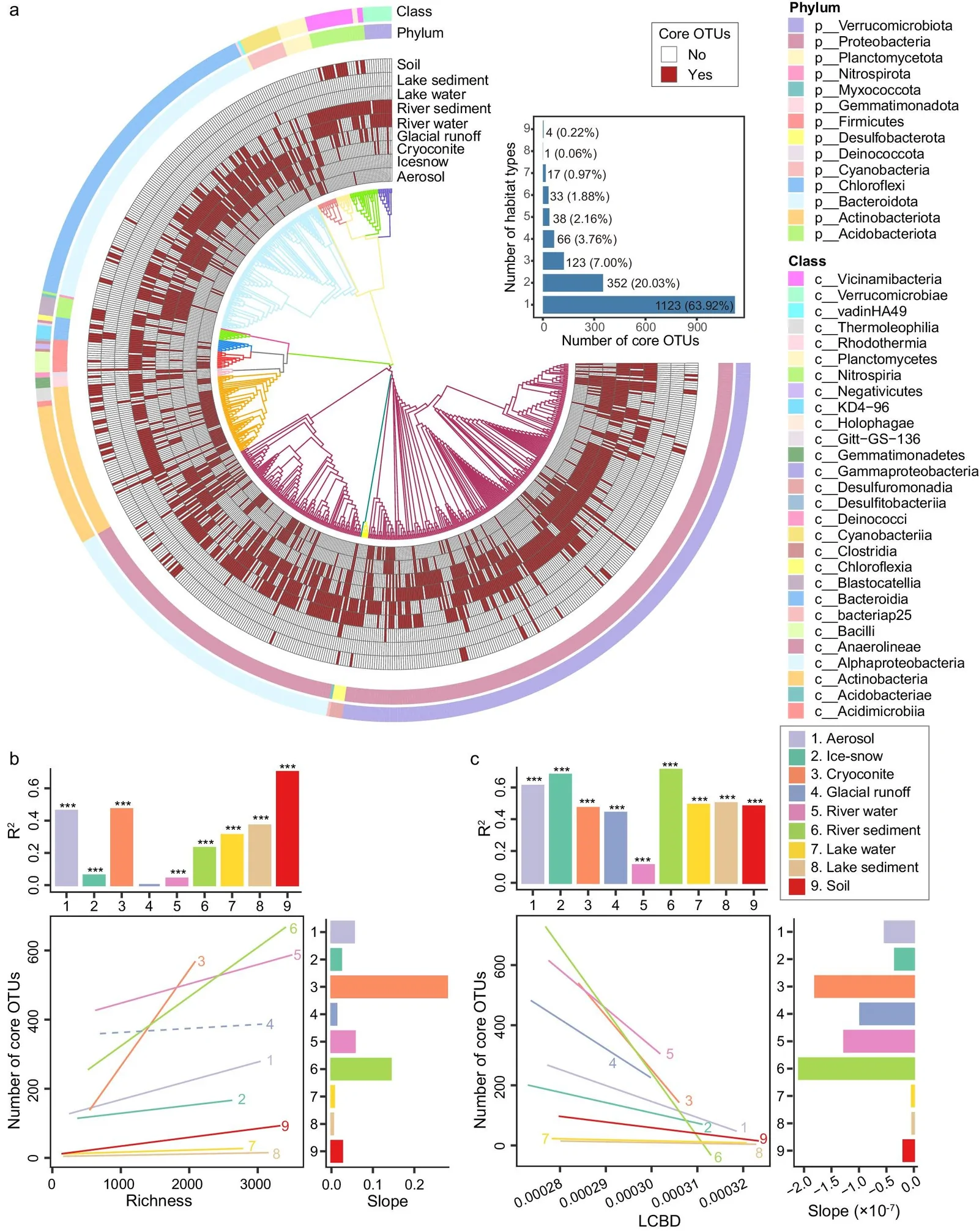
Distribution of core OTUs across 9 habitat types. (a) Phylogenetic tree of core OTUs at the phylum and class levels. The heatmap indicates whether these OTUs are core OTUs of the corresponding habitat, with red indicating ‘yes’ and white indicating ‘no’. Outer rings represent the classification of these OTUs at the phylum and class level. The bar chart in the upper-right corner shows the distribution of all core OTUs across the nine habitat types. (b) Linear regression relationships between the number of core OTUs and bacterial richness for each habitat. (c) Linear regression relationships between the number of core OTUs and community uniqueness (LCBD) for each habitat. Statistically significant relationships are shown with solid lines (*P* < 0.05), while dashed lines show non-significant relationships (*P* ≥ 0.05). The top panel displays the values of R^2^ for relationships between the number of core OTUs and bacterial richness or LCBD, and the right panel shows slopes of the ordinary least-squares regression lines fitted to these relationships.

The number of core OTUs and their contribution to total abundance varied significantly among habitats, generally consistent with the high beta-diversity among them. River sediment (710 core OTUs) and river water (701) had the highest core OTU number, followed by cryoconite (520), glacial runoff (509), aerosol (254), ice-snow (185), soil (95), lake water (23), and lake sediment (14) (Fig. S14). The percentage of core OTUs in the total OTUs was highest in glacier runoff (0.4%) and lowest in lake sediment (0.005%). Despite their small share of richness, core OTUs accounted for more than 55% of the relative abundance in glaciers, including ice-snow (58.1%), cryoconite (57.5%), and glacial runoff (61.9%), but merely 3.1% and 7.8% in the lake sediment and soil, respectively (Fig. S14).

Across habitats, the number of core OTUs increased with richness and decreased with community uniqueness (LCBD), indicating that more diverse and compositionally less distinct communities tend to harbor more core taxa (Fig. 5b and c). Except for glacial runoff, the number of core OTUs increased significantly with richness in eight habitats. The coefficient of determination (R^2^) for the relationship between the number of core OTUs and richness was high in soil (0.70), cryoconite (0.47), and aerosol (0.46). In cryoconite, river sediment, and river water, the number of core OTUs was particularly sensitive to changes in richness. Conversely, the number of core OTUs was significantly and negatively correlated with LCBD across all habitats, with R^2^ > 0.4 in all habitats except river water. In cryoconite and river sediment, the number of core OTUs was particularly sensitive to LCBD, suggesting that communities with fewer core taxa tend to be more compositionally distinct. These results indicate that core microbiomes differed markedly among habitats: most core OTUs are restricted to a single habitat, and only four OTUs are shared across all nine habitats.

### The influence of climate factors on microbial communities

Linear mixed-effects models (LMMs) were employed to assess the effects of annual mean temperature (AMT), annual mean precipitation (AMP), annual mean solar radiation (AMS), and their interactions on bacterial richness (Richness) and LCBD. Regression coefficients represent both the directions and magnitudes of community response, with habitat type modeled as a random intercept. Conditional R^2^ (richness: 0.448; LCBD: 0.422) was much higher than marginal R^2^ (richness: 0.098; LCBD: 0.160) for richness and LCBD (Fig. 6a and c), indicating that differences among habitat types strongly modulate microbial responses to climate. Among fixed effects, AMP was the most significant factor affecting both richness and LCBD, followed by AMT. The effect sizes of AMP and AMT were significantly larger than those of AMS, as well as the joint effects of two or three factors. Notably, AMP and AMT showed opposing effects on richness and LCBD for all five ecosystems. The interaction between AMT and AMP was significant for both response variables, with a negative joint effect on richness and a positive joint effect on LCBD (Fig. 6a and c), suggesting that combined warming and increasing precipitation might reduce bacterial richness while enhancing the compositional uniqueness of communities. In contrast, the three-way interaction among AMT, AMP, and AMS had relatively weak effects, explaining only 3.4% of the explained variation in richness and 4.9% of the explained variation in LCBD (Table S4).

**Figure 6.**
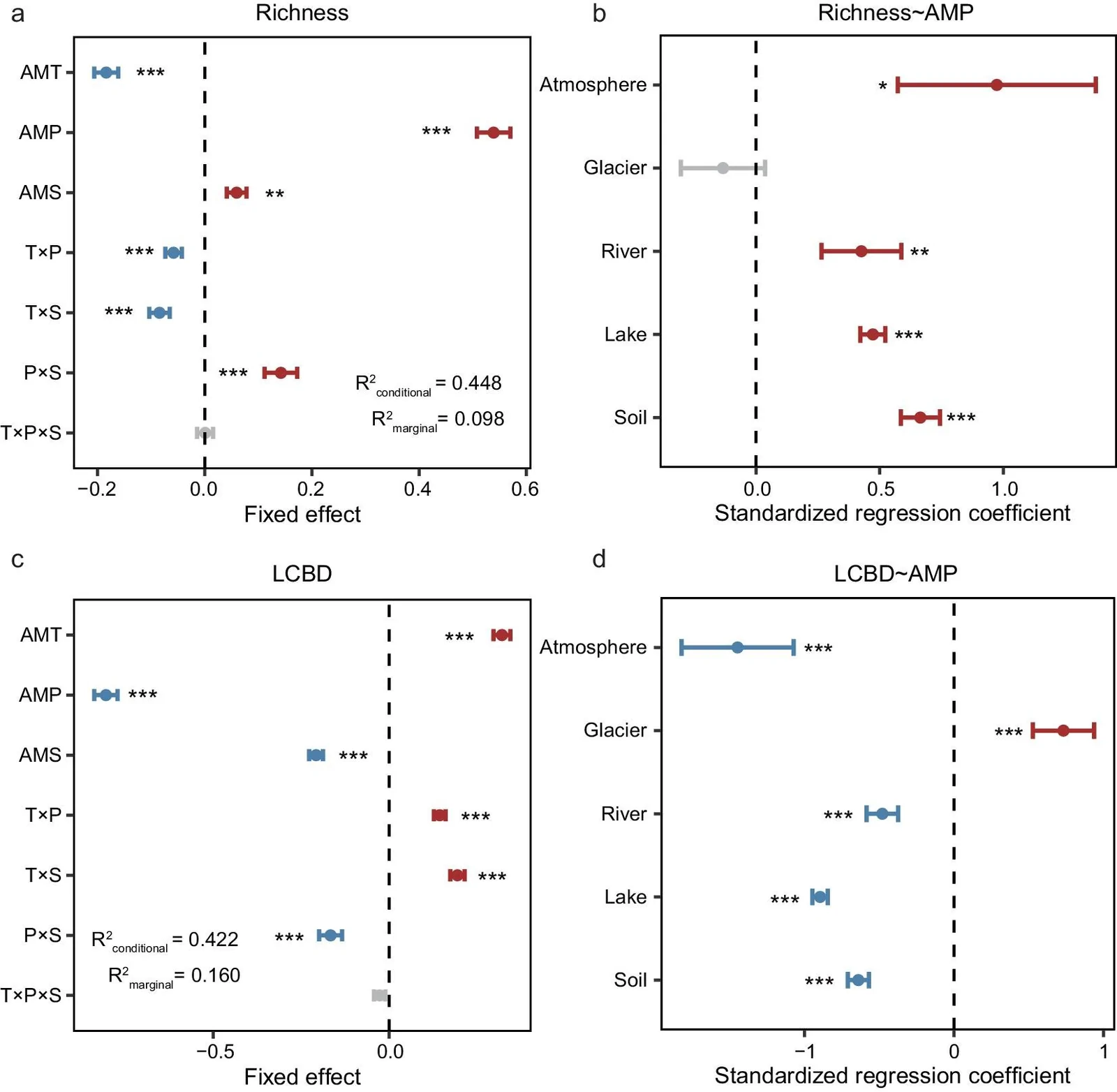
Effects of climatic variables on bacterial communities. (a) Effects of climatic variables on bacterial richness by a LMM. (b) The effects of AMP on bacterial richness in each ecosystem using MLRs. (c) Effects of climatic variables on bacterial LCBD using a LMM. (d) Effects of AMP on LCBD across ecosystems using MLRs. In LMMs, AMT, AMP, and AMS and their interactions were treated as fixed effects, while habitat type was termed as a random intercept (*y* ∼ AMT × AMP × AMS + (1 | habitat type)). The panel shows the coefficients (dots) and 95% CIs (bars) for a LMM, illustrating the fixed main and interaction effects of AMT, AMP, and AMS on bacterial richness and LCBD. All the predictors were standardized. T × P: the interaction of AMT and AMP; T × S: the interaction of AMT and AMS; P × S: the interaction of AMP and AMS; T × P × S: the interaction of AMT, AMP, and AMS. In MLRs, the panel shows coefficients (dots) and 95% CIs (bars) for AMT, AMP, AMS, and their interaction on bacterial richness and LCBD, with standardized effects. All predictors were standardized before conducting MLR.

AMP emerged as the strongest positive driver of bacterial richness, accounting for 66.8% of the explained variation in richness (Fig. 6a and Table S4). To examine ecosystem-specific responses to AMP, we fitted multiple linear regression (MLR) within each ecosystem (Fig. 6b). Richness increased significantly with AMP in the atmosphere, river, lake, and soil ecosystems, but the correlation was not significant in the glacier ecosystem. Atmospheric bacteria showed the strongest positive response to AMP (Fig. 6b), followed by river, lake, and soil communities.

AMP was also the most significant factor influencing LCBD, accounting for 54.6% of the total explained variation, followed by AMT (14.0%) and AMS (8.4%) (Fig. 6c and Table S4). At the plateau scale (pooling habitats), the effect of AMP on LCBD was approximately threefold that of AMT (Fig. 6c). Ecosystem-specific MLR analyses indicated that AMP increased LCBD in glaciers but decreased LCBD in the atmosphere, rivers, lakes, and soils. Again, LCBD in the atmosphere was the most responsive to AMP (Fig. 6d). These opposing responses indicate that increased precipitation tends to homogenize communities in most ecosystems, while simultaneously enhancing the uniqueness of glacial communities. Microbial responses to climate factors and the joint effects of AMP, AMT, and AMS are detailed in Supplementary Results R8.

## DISCUSSION

### Dispersal links a highly connected multi-sphere bacterial metacommunity on the Tibetan Plateau

Our continental-scale survey reveals that bacterial communities inhabiting the atmosphere, glaciers, lakes, rivers, and soils of the Tibetan Plateau constitute a highly connected metacommunity, characterized by extensive OTU overlap but a relatively small number of ecosystem-specific taxa. More than 55% of OTUs were detected in at least two ecosystems, and nearly 6% were shared by all five ecosystems, indicating that none of these spheres is microbiologically isolated. The strong cross-ecosystem sharing of OTUs along the atmosphere-glacier-downstream systems supports the view that microbial dispersal links seemingly disparate compartments of the ‘Asian water tower’ into a single, coupled microbial landscape [2].

The atmosphere, primarily via aerosol transport and deposition, appears to represent a significant regional source of glacier and downstream microbiota. In our dataset, atmospheric OTUs accounted for 89.5% of the total abundance of OTUs detected in glaciers, consistent with long-range atmospheric transport as a key mechanism seeding supraglacial microbial communities, as reported for glacier and snow ecosystems in other mountain and polar regions [29]. Moreover, OTUs shared between glaciers and downstream systems, such as rivers, lakes, and soils, account for more than 80% of the total abundance of glacier microbes, indicating that a substantial component of glacier-derived bacteria may convey microbial functions and traits into riverine, lacustrine, and terrestrial environments [18,30].

Aerosols are major vectors for atmospheric transport of microorganisms, including bacteria, fungi, and viruses [31]. Consistent with global patterns, the aerosol microbiome on the plateau harbors a distinct composition, with radiation-tolerant Firmicutes being particularly abundant (Fig. 3; [32]). In our dataset, the responses of aerosol microbial richness and community composition to AMP were the strongest among the five ecosystems (Fig. 6), indicating tight coupling between atmospheric hydrometeors and airborne microbiota. The sources of aerosols, including natural emitters such as deserts, soils, and vegetation, and anthropogenic emissions, are key determinants of their microbial composition, while transport pathways impose additional filtering that can selectively convey microorganisms across regions [31,33]. Therefore, aerosol microbiomes may serve as biological tracers of the westerlies, the Asian monsoon, local circulations over plateaus, and, more broadly, of climate change and human activities [32–34].

Exotic microorganisms can attach to mineral dust or organic particles and be transported by regional and large-scale atmospheric circulation, resulting in distinct microbial population distributions on glacier surfaces [35,36]. More than 90% of atmospheric bacterial abundance was detected in glacial environments, including ice-snow, cryoconite, and glacier runoff (Fig. 2b), reinforcing the strong connection between atmospheric and glacial microbes. Ecosystems surrounding the Tibetan Plateau, including urban, meadow, forest, and desert regions, are therefore likely to represent important source pools for glacial microorganisms. Dust and bioaerosol deposition onto snow and ice can reflect contributions from both regional deserts and distant source regions. For example, a substantial fraction of glacier bacteria may originate from the Taklimakan Desert and other arid regions, transported by the westerlies, while additional lineages are delivered by monsoon-related air masses and surrounding ecosystems [32,34,35,37]. Although the aerosol appears to be the most likely source of bacteria in terrestrial surface systems, we cannot rule out reverse transport from glaciers, lakes, or soils to the atmosphere; these habitats may also occasionally serve as sources of airborne bacteria.

Glacier-derived microbes detected in downstream ecosystems accounted for more than 80% of the total relative abundance in glacial habitats and remained abundant in rivers, lakes, and soils, where they contributed 75.8%, 59.8%, and 63.8% of the total relative abundance, respectively (Fig. 2b), confirming that the glacier-derived microbes constitute an important source pool for downstream microbial communities [38]. This pattern indicates strong microbial connectivity between the glacier and downstream ecosystems, likely mediated by meltwater runoff, suspended particles, and hydrological transport [18]. The higher proportion in rivers is consistent with their direct hydrological connection to glacier meltwater, whereas the lower proportions in lakes and soils suggest additional effects of dilution, sedimentation, residence time, and local environmental selection. Our evidence for both direct and indirect pathways of microbial transport from glaciers to lakes (Fig. S4) further supports the idea that glacier-associated microorganisms can be redistributed across downstream ecosystems. However, because shared taxa do not necessarily indicate active colonization or ecological function, these results should be interpreted as evidence of potential microbial transport and connectivity rather than definitive proof of glacier-derived microbial establishment.

This pronounced multi-sphere connectivity, evidenced by extensive atmosphere-glacier-downstream OTU sharing and a low proportion of strictly ecosystem-specific taxa, is consistent with metacommunity theory, which defines the metacommunity as a set of local communities linked by the dispersal of multiple, potentially interacting species [28]. Accordingly, the small proportion of strictly unique OTUs in each ecosystem suggests that dispersal is frequent enough to prevent complete isolation, even under extreme environmental conditions. Although this study systematically compared four potential transport scenarios (Fig. S3) and clarified that atmospheric–glacier–downstream transport is the most likely mode of microbial cross-sphere migration in this region, an analytical framework based on a single dominant mode still struggles to fully reconstruct the complex dynamics of multiple processes acting in concert in natural environments. In forthcoming work, a probabilistic simulation framework is needed to couple multiple modes to quantify contributions from various transport pathways and to refine further cross-sphere microbial transport models that more realistically reflect natural processes.

### Habitat filtering and core microbiome structure bacterial biogeography across spheres

Despite intense atmosphere–glacier–downstream connectivity, when we considered the relative abundance of OTUs, we found that shared taxa were rare or transient in some habitats but dominant in others. Thus, heterogeneity in the relative abundance of OTUs across habitats could reflect distinct community assembly mechanisms underlying current habitat differentiation in bacterial community composition, diversity, and spatial distribution.

Glacier-associated habitats (ice-snow, cryoconite, and glacial runoff) are dominated by Proteobacteria and Bacteroidota, reflecting cold, oligotrophic, and hydrologically dynamic conditions that favor metabolically versatile heterotrophs and copiotrophs [39,40]. River and lake waters likewise share high contributions of Proteobacteria and Bacteroidota, whereas lake waters are additionally enriched in Actinobacteriota, a hallmark of many freshwater ecosystems [41,42]. In contrast, soils and river sediments have the highest richness and are enriched in typical edaphic and sediment-associated lineages such as Acidobacteriota and Chloroflexi. These habitat-specific taxonomic signatures suggest that temperature regimes, substrate supply, hydrological disturbance, and redox conditions jointly impose strong environmental filtering across spheres.

Diversity metrics showed that habitats differed not only in richness but also in their contribution to regional beta diversity. River sediments and soils were the richest habitats, whereas species-poor lake waters and ice-snow exhibited high LCBD values, indicating that they contribute disproportionately to regional compositional turnover. This decoupling between α-diversity and community uniqueness underscores that species-poor habitats can exert an outsized influence on regional beta diversity. That richness alone is insufficient for assessing ecological importance or vulnerability [43]. Spatial distance–decay patterns add a biogeographic dimension: significant distance–decay occurs in eight of nine habitats, with the steepest rates in cryoconite and glacial runoff, suggesting that both dispersal limitation and spatially structured environmental gradients constrain community similarity in glacier-associated environments. After controlling for environmental dissimilarity using partial Mantel analyses, geographic distance–decay of bacterial community similarity remained significant in six of the nine habitats, whereas ice-snow, river water, and river sediments showed no significant distance–decay (Fig. S17). The lack of a significant distance–decay relationship in ice-snow may reflect its direct exposure to atmospheric deposition, episodic snowfall, wind redistribution, and air-mass transport, which can repeatedly introduce microorganisms from local to long-distance sources, thereby obscuring simple geographic signals [44–46]. In river water and river sediment, strong hydrological connectivity and downstream transport may similarly weaken the independent effect of geographic distance [47,48].

The low proportion of core OTUs in the total bacterial community and their differing contributions to total relative abundance across habitats may reflect distinct assembly mechanisms in bacterial communities (Fig. S14). Using an abundance-prevalence criterion, we identified only 0.005%–0.4% of OTUs within each habitat as core members. In ice-snow, cryoconite, glacial runoff, and river water, core OTUs accounted for more than 50% of the total relative abundance, suggesting that these habitats were dominated by a small set of abundant and frequently detected taxa. Greater atmospheric or hydrological connectivity in those habitats may facilitate homogenizing dispersal and weaken dispersal limitation by repeatedly introducing and redistributing microbial taxa, while environmental selection imposed by habitat-specific physicochemical conditions may consistently favor the retention and persistence of a relatively small set of taxa well adapted to each habitat [49]. In contrast, core OTUs contributed <10% of the total relative abundance in lake sediments and soils, suggesting that these habitats were less dominated by broadly distributed taxa and instead featured greater contributions from locally abundant or spatially restricted taxa. This may reflect a greater contribution of environmental selection and dispersal limitation to bacterial assembly in lake sediments and soils [50,51].

Glacier ecosystems illustrate how habitat filtering and core microbiomes interact. Core OTUs in ice-snow, cryoconite, and glacial runoff accounted for 0.139%, 0.31%, and 0.363% of the total OTUs in their respective habitats, whereas core OTUs shared by ice-snow, cryoconite, and glacial runoff represented only 0.007% of the OTU pool in the glacier ecosystem (Fig. S14). Together with the high relative abundance of cores in each glacier habitat (more than 55%), this indicates that a relatively small set of taxa underpins most of the biomass and potential functions within glacier ecosystems (Fig. S14 and Fig. 5). Among all the nine habitats, cryoconite and glacial runoff shared the largest number of core OTUs (104 OTUs), dominated by Gammaproteobacteria (50.9%), and Bacteroidia (27.9%), with *Polaromonas* (30.8%), *Ferruginibacter* (13.5%) as prominent genera. Many of these genera have also been reported in other cryosphere ecosystems [52], underscoring their competitive advantage and functional importance in low-temperature, oligotrophic environments.

Across habitats, the number of core OTUs increased with richness but decreased with LCBD, particularly in soils, cryoconite, and aerosols, suggesting that compositionally common communities harbor larger sets of core taxa and thus greater functional redundancy, whereas compositionally unique habitats such as lake waters rely more on non-core and rare lineages. These patterns support a framework in which niche processes (environmental filtering, habitat specialization) primarily structure dominant taxa, whereas the rare biosphere is more strongly shaped by near-neutral dynamics [53].

### Precipitation as the dominant climatic driver of bacterial diversity and uniqueness

Climate emerges as a key regulator of bacterial communities, with mean annual precipitation exerting a much stronger effect on richness and community uniqueness than mean annual temperature or sunshine duration. Three non-mutually exclusive mechanisms of precipitation might play in structuring multi-sphere microbiomes: (i) controlling the amount and timing of liquid water, a primary constraint on microbial activity and dispersal; (ii) modulating hydrological and depositional connectivity among habitats; and (iii) shaping resource supply and disturbance regimes along the atmosphere–glacier–downstream continuum.

First, precipitation was the strongest positive driver of bacterial richness, particularly in the atmosphere, where a one-standard-deviation increase in AMP corresponded to a nearly one-standard-deviation increase in richness. Positive effects of AMP on richness were also observed in rivers, lakes, and soils. Mechanistically, higher precipitation enhances the formation of cloud droplets and raindrops that scavenge and concentrate bioaerosols [54,55], enhances wet deposition of microorganisms and nutrients onto glaciers and terrestrial surfaces, and sustains surface and subsurface flow paths that connect soils, streams, and lakes [17]. These processes jointly increase the number of sources and colonization opportunities for microbes, broaden realized niche space, and buffer against local extinction, thereby elevating richness in many habitats.

In contrast, bacterial richness in glaciers showed no significant response to AMP. We interpret this apparent insensitivity as evidence that glacier microbiomes are primarily governed by local cryosphere controls—such as temperature near the melting point, freeze-thaw cycles, light regime, and the availability of cryoconite and weathering crust microhabitats—rather than by regional precipitation totals [56,57]. Strong selection by low temperature, oligotrophy, and episodic melt likely favors a relatively narrow set of stress-tolerant, metabolically flexible taxa, limiting the extent to which additional precipitation can translate into higher richness. This is consistent with our finding that a small but abundant core microbiome dominates glacier habitats. The lack of an AMP-richness relationship in glaciers, in contrast to positive associations elsewhere, suggests that local habitat constraints can override regional climatic forcing in ice-snow, cryoconite, and glacial runoff, and that these constraints can also override the functional repertoire required for their persistence in an ecosystem with similar environmental constraints [52]. Furthermore, ongoing climate change may strengthen associations among habitats within glacier ecosystems by accelerating physical and biogeochemical reorganization. Mountain glaciers have retreated rapidly over recent decades [58], with cascading landscape transformations driven by changes in sediment delivery, hydrological routing, and weathering processes [59]. Microorganisms have the potential to serve as ecological and hydrological indicators, with potential utility for tracing hydrological paths, exploring the heterogeneity of subglacial habitats, and monitoring changes in glacial watersheds over time [26].

Second, AMP exerted contrasting effects on LCBD across ecosystems, revealing a dispersal-selection trade-off in how precipitation regulates beta diversity. In glaciers, increased precipitation was associated with higher LCBD, indicating that glacier communities became more compositionally unique as AMP increased. One plausible mechanism is that greater precipitation, integrating distinct microbial atmospheric fetches and source regions, accentuates differences among glaciers, thus enhancing among-glacier heterogeneity in bioaerosol inputs, snow accumulation, and melt histories [60–62]. In the atmosphere, rivers, lakes, and soils, however, higher AMP was associated with lower LCBD, suggesting that precipitation tends to homogenize communities by strengthening hydrological and depositional connectivity, mixing previously isolated assemblages, and smoothing local environmental contrasts. This pattern has also been reported in other fluvial and lacustrine systems where increased discharge and mixing reduce spatial turnover [63].

Third, precipitation does not operate in isolation but interacts with other climatic and geomorphic drivers to shape microbial landscapes. Monsoon intensity and the frequency of extreme precipitation events can regulate flood pulses, turbidity, and sediment loads, thereby altering light regimes, substrate supply, and disturbance in glacier-fed streams and downstream lakes [64,65]. In such systems, intermediate disturbance may promote coexistence by limiting competitive exclusion, whereas very frequent or intense events can lead to community resets, favoring fast-growing opportunists [66,67]. The ecosystem-specific responses we observe—precipitation-driven gains in richness and homogenization in downstream habitats versus increased compositional uniqueness in glaciers—are therefore consistent with a climate-mediated balance among dispersal, disturbance, and environmental filtering across spheres.

Taken together, these results suggest that projected changes in the Asian monsoon and hydrological regimes could substantially reshape both the magnitude and spatial structure of microbial diversity on the plateau. Enhanced precipitation may increase atmospheric and downstream richness while promoting microbial homogenization, whereas glacier communities may become more idiosyncratic as snow accumulation and melt input regimes diverge among catchments. Such shifts could alter the balance between community homogenization and differentiation across spheres, with cascading consequences for microbe-mediated biogeochemical processes and water quality in the ‘Asian water tower’ [2,3]. Integrating climate projections with high-resolution microbial, hydrological, and biogeochemical observations will therefore be critical for anticipating how precipitation-driven changes propagate through the atmosphere-glacier-downstream continuum and for using microbial communities as sensitive sentinels of ongoing changes.

## CONCLUSIONS

Our plateau-wide synthesis portrays the Tibetan Plateau as a climate-sensitive, multi-sphere bacterial metacommunity in which atmospheric dispersal, glacier filtering, and downstream hydrological routing jointly connect microbial life in air, ice, water, and soils. Strong habitat filtering and the architecture of core microbiomes impose distinct biogeographic and phylogenetic fingerprints on each sphere, whereas precipitation emerges as the dominant climatic driver regulating richness, community uniqueness, and the balance between homogenization and differentiation across ecosystems. By integrating multi-habitat biodiversity data with climate gradients, this study provides a unifying framework for how dispersal, habitat filters, core taxa, and precipitation-driven dynamics together structure bacterial communities across the spheres of the ‘Asian water tower’. In this context, bacteria form an invisible yet highly dynamic network that links the cryosphere, hydrosphere, and terrestrial ecosystems. Incorporating this microbial dimension into assessments of the ‘Asian water tower’ will be essential for anticipating how climate-driven changes in glaciers and hydrology cascade through regional biodiversity, biogeochemical cycling, and water-resource security.

## MATERIALS AND METHODS

### Samples and sequence data collection

A total of 2624 samples from the Tibetan Plateau, covering five distinct ecosystems: atmosphere, glaciers, rivers, lakes, and soils (Fig. 1), were collected and sequenced by the Second Tibetan Plateau Scientific Expedition and Research Program (STEP) from 2017 to 2023. In addition, 749 previously published data were included in the analysis (Table S1). The communal catalogue comprises 3373 amplicon sequencing data for the V4–V5 hypervariable regions of the bacterial 16S rRNA gene, using the polymerase chain reaction (PCR) primers 515F (5′-GTGCCAGCMGCCGCGGTAA-3′) and 907R (5′-CCGTCAATTCMTTTRAGTTT-3′). The samples cover nine habitat types, including aerosol (*n* = 178), ice-snow (*n* = 241), cryoconite (*n* = 257), glacial runoff (*n* = 132), river water (*n* = 363), river sediment (*n* = 197), lake water (*n* = 715), lake sediment (*n* = 516), and soil (*n* = 774). The newly sequenced data were deposited in the NCBI SRA database with project accession number PRJNA1233668. Detailed procedures for sampling, DNA extraction, and sequencing data processing are provided in the Supplementary Methods M1.

### Climatic data collection

Climatic factors, including AMT, AMP, and AMS, were extracted based on the geographic locations of each sampling site using the WorldClim version 2.1 dataset (http://www.worldclim.org) [68]. Detailed information is described in Supplementary Methods M2.

### Data analyses

#### Microbial connectivity among ecosystems on the Tibetan Plateau

Potential interactions among five ecosystems were analyzed using OTU overlap. Detailed analyses and visualization procedures are described in Supplementary Methods M3.1.

#### Bacterial alpha and beta diversity

The alpha-diversity indices, such as richness, Shannon–Wiener, Chao1, and evenness, were calculated, and Wilcoxon tests were conducted to assess variation in alpha-diversity across pairwise habitats. The details for the analyses are provided in Supplementary Methods M3.2.

NMDS and PERMANOVA analyses were used to assess the significance of compositional differences among habitats. LCBD were analyzed, and pairwise LCBD comparisons were conducted using Wilcoxon tests. Linear and quadratic models were applied to identify the best fit between LCBD and Richness, as indicated by the lowest Akaike information criterion. The distance decay of Bray–Curtis similarity was examined for each habitat. The details are provided in Supplementary Methods M3.3. In addition, the characteristics of co-occurrence networks for each habitat were analyzed (Supplementary Methods M3.4).

#### Bacterial community composition and the core OTUs for each habitat

The bacterial community composition, core OTUs for each habitat, and the overlap of core OTUs across habitats were analyzed. Identification criteria for core OTUs and additional analyses are provided in the Supplementary Methods M3.5.

#### The impact of climate factors on bacterial communities

LMMs were used to assess the effects of AMT, AMP, and AMS on bacterial Richness and LCBD (Supplementary Methods M3.6 and M3.7).

## Supplementary Material

nwag463_Supplemental_File

## Data Availability

Raw sequence data are available in the NCBI Sequence Read Archive under accessions of PRJNA1233668, PRJNA1056829, PRJNA1013198, PRJNA608087, PRJNA790017 and PRJNA980427.
