# Supplementary material for: A climate-sensitive, contemporary multi-sphere bacterial metacommunity across the Tibetan Plateau
Source: Natl Sci Rev. 2026 Aug 3;13(17):nwag463. doi: 10.1093/nsr/nwag463 (PMC13639186; doi:10.1093/nsr/nwag463)
Supplement: Supplementary file 1 [file nwag463_supplemental_file.docx]

**Supplementary Information for**

**A climate-sensitive, contemporary multi-sphere bacterial metacommunity across the Tibetan Plateau**

Yongqin Liu et al.,

The file includes:

Supplementary Methods (M1-M3)

Supplementary Results (R1-R8)

Supplementary Tables (S1-S4)

Supplementary Figures (S1-S17)

**Supplementary Methods**

**M1 Sample collection and sequencing data processing**

We followed the same protocols for collecting samples from each habitat as those used in previous studies [1-6]. Microbial DNA was extracted using the FastDNA Spin Kit (MP Biomedicals, Santa Ana, CA) or the PowerSoil DNA Isolation Kit (QIAGEN) according to the manufacturer’s instructions. Bacterial 16S rRNA genes were amplified by polymerase chain reaction (PCR) and assessed using the Illumina MiSeq platform, as previously described [1,4]. We employed the 515F and 907R primer set (515F: 5’-GTGCCAGCMGCCGCGGTAA-3’; 907R: 5’-CCGTCAATTCMTTTRAGTTT-3’) to amplify the V4-V5 hypervariable regions of the 16S rRNA gene.

Raw reads for each sample were filtered to remove adapters and low-quality sequences by “Trimmomatic” (version 0.33, http://www.usadellab.org/cms/?page=trimmomatic, parameters: SLIDINGWINDOW: 4: 15 MINLEN:75) [7]. The paired-end sequences were merged by “FLASH” (https://ccb.jhu.edu/software/FLASH/) with default parameters. Reads that could not be merged were discarded [8]. Then “cutadapt” was applied to trim the primers for each data set with the following parameters: “-g forward_primer -G reverse_primer --trimmed-only” [9]. The remaining sequences were clustered into operational taxonomic units (OTUs) with a 97% similarity cutoff [10] using VSEARCH (https://github.com/torognes/vsearch) [11], and chimeric sequences were identified and removed using UCHIME (http://drive5.com/uchime/uchime_download.html) [12]. Finally, the phylogenetic affiliation of each 16S rRNA gene sequence was analyzed using the UCLUST algorithm (http://www.drive5.com/usearch/) against the SILVA (SSU138.1) 16S rRNA database, with an 80% confidence threshold [13]. All singletons and OTUs assigned to mitochondrial and chloroplast sequences were discarded.

**M2 Climatic data collection**

Climatic factors, including the annual mean temperature (AMT), the annual mean precipitation (AMP), and the annual mean solar radiation (AMS), were extracted as 1970-2000 average values based on geographic locations (i.e., latitude and longitude) of each sampling site using the WorldClim version 2.1 dataset (http://www.worldclim.org) [14]. All climatic information was obtained at a spatial resolution of 30 arc-seconds, approximately 1 km per grid cell.

**M3 Data analyses**

**M3.1 Microbial connectivity among ecosystems on the Tibetan Plateau**

We explored potential interactions across five ecosystems by analyzing OTU overlap and visualizing the results with an UpSet plot generated in the R package “UpSetR” [15]. The following ratios were examined: the proportion of OTUs shared between any two ecosystems (n1,i-j) relative to the total number of OTUs (N) (AAi-j%=n1,i-j/N×100), the proportion of OTUs in an ecosystem (n2,i) relative to N (BBi%=n2,i/N×100), the proportion of OTUs restricted to an ecosystem (n3,i) relative to N (CCi%=n3,i/N×100), and the proportion of OTUs shared among five ecosystems (n4) relative to N (DD%=n4/N×100). Here, i is one of the five ecosystems, and j is one of the remaining four ecosystems. The percentage of OTUs shared between two ecosystems (AAi-j%) was used to measure their potential interconnection. In addition, OTU overlap across habitats within an ecosystem was analyzed using the same strategy, with 100% representing the total number of OTUs in the ecosystem.

Four conceptual models were constructed, with the glacier, soil, lake, and river ecosystems serving, respectively, as central nodes in multi-sphere interconnections (i.e., intermediate systems), assuming that microbial transport occurs via atmospheric deposition to the intermediate systems and then to other ecosystems. Using the scenario in which the glacier ecosystem serves as the intermediate system as an example, the mean relative abundance of OTUs shared between the atmosphere and the glacier ecosystem (FF1%) or between the atmosphere and the glacier ecosystem (FF2%) was used to reflect the intensity of atmospheric influence on glacier-surface microbial communities. Similarly, the mean relative abundance of OTUs shared among the atmosphere, glaciers, and downstream ecosystems in glaciers was used to quantify the intensity of microbial transport from glaciers to these ecosystems (GGi%). The mean relative abundance of OTUs shared among atmosphere, glaciers, and downstream ecosystems was used to quantify the strength of links between glaciers and a downstream ecosystem (EEi%). Here, i is one of the three ecosystems, e.g., rivers, lakes, and soils. Two quantitative metrics were used to compare the likelihood of four microbial transmission pathways: 1) the ratio of OTUs shared between the atmosphere and the intermediate system to the total abundance of OTUs in the intermediate system (FF2%); and 2) the sum of the ratios of OTUs shared between each of the three downstream systems and both the intermediate system and the atmosphere to the total abundance of OTUs in the intermediate system (GG%). The scenario with higher values of both indicators was selected as the most ideal.

Additionally, we analyzed the potential for glaciers to influence lake microbial composition via rivers. Using the atmosphere-glacier-downstream system as the microbial transport pathway, we classified the OTUs detected in the lake ecosystem into four categories: (ⅰ) OTUs occurring in lakes but absent from both the glaciers and rivers; (ⅱ) OTUs shared between glaciers and rivers, and also occurring in lakes; (ⅲ) OTUs shared between rivers and lakes but absent from glaciers; and (ⅳ) OTUs shared between glaciers and lakes but absent from rivers. Among these, category (ⅱ) was identified as capable of being transported from glaciers to lakes via rivers and was termed “indirectly transported OTUs”, whereas category (ⅳ) was identified as those that could be transported directly from glaciers to lakes and was termed “directly transported OTUs”. When only considering the presence and absence of OTUs, two indicators were adopted to evaluate the potential of glaciers to shape lake microbial composition via river pathways: the proportion of indirectly transported OTUs relative to all OTUs detected in glaciers, and the proportion of directly transported OTUs relative to all OTUs detected in glaciers. When incorporating OTU abundance into the analysis, two pathways were used to assess the potential influence of glaciers on lake microbial composition: the average relative abundance of indirectly transported OTUs via rivers and that of directly transported OTUs to lakes.

**M3.2 Bacterial alpha diversity in each habitat**

We calculated five alpha diversity indices, including bacterial richness, evenness, Shannon-Wiener diversity, Simpson index, and Chao1. To control for potential biases arising from variation in sequencing depth or sample size, the OTU matrix was subsampled to 5000 reads per sample before calculating alpha diversity. The raw OTUs matrix was retained for other analyses. Wilcoxon tests were used to assess differences in alpha diversity between pairs of habitats. Additionally, non-metric multidimensional scaling (NMDS) based on the Bray-Curtis similarity index was performed to examine variation in community structure across habitats. Permutational analysis of variance (PERMANOVA) was used to assess the significance of community differences in different habitats [16].

A non-asymptotic approach based on standardized sample size was used to infer bacterial richness using the R package “iNEXT” [17]. Rarefaction and extrapolation curves were constructed using a standardized sample size to assess whether the retained reads were sufficient to cover the vast majority of species and to estimate theoretical richness.

**M3.3 Beta diversity for bacterial communities and distance-decay pattern**

We calculated local contributions to beta diversity (LCBD) [18] and compared LCBD between pairs of habitats using Wilcoxon tests. High LCBD values indicate the site's great importance in explaining the observed total variation in species composition across sites [19]. That is, from an ecological perspective, LCBD values represent the degree of uniqueness of sampling units within the metacommunity. Linear and quadratic models were applied to analyze the relationship between LCBD and bacterial richness, and the most appropriate model was selected based on the Akaike information criterion (AIC) [20].

To explore spatial distance-decay relationships for each habitat, Bray-Curtis dissimilarities were calculated among samples within each habitat, and Mantel tests were then performed to examine the relationship between geographic distance and Bray-Curtis dissimilarities, using Spearman's rank correlation and 9999 permutations. The Mantel r-value was calculated to assess the strength of the distance-decay relationship. The slopes of the ordinary least-squares regression line fitted to the relationship between the spatial distance and community dissimilarity were applied as the rate of distance decay. To further evaluate whether geographic distance had an independent effect on bacterial community dissimilarity after accounting for environmental differences, partial Mantel tests were additionally performed for each habitat. For each habitat, environmental dissimilarity matrices were calculated using the available standardized environmental variables and Euclidean distance. Partial Mantel tests were then conducted between the Bray-Curtis community dissimilarity matrix and the geographic distance matrix while controlling for the corresponding environmental dissimilarity matrix. Spearman’s rank correlation and 9999 permutations were used to assess the significance of the partial Mantel correlations. The resulting partial Mantel r-values were used to quantify the independent association between geographic distance and bacterial community dissimilarity after accounting for environmental dissimilarity.

To avoid the potential biases introduced by varying sample sizes among habitats, the following approach was employed. For each habitat, 100 samples were randomly selected, and the mean bacterial richness and LCBD were calculated. This resampling and averaging procedure was independently replicated 1000 times, generating a dataset of 1000 mean values for each habitat. Nonparametric Wilcoxon tests were then used to assess significant differences in microbial diversity across habitats using this resampled dataset. This approach ensured that the comparison was robust to variations in original sample sizes across habitats.

**M3.4 Characteristics of the co-occurrence network for each habitat**

We constructed co-occurrence networks for all microbial communities across all habitats using the OTU relative abundance matrix derived from the raw, unrarefied OTU count matrix. To minimize the impact of varying sample sizes across habitats on network construction, 100 samples were randomly selected without replacement from each habitat 100 times. All networks were constructed using Spearman correlations among abundant OTUs (average relative abundance > 0.001%) from random samples. Spearman correlations between OTUs were calculated, and only confident connections [correlation coefficient (ρ) > 0.7 or < -0.7, *P* < 0.05] were retained. Additionally, we calculated the number of nodes and connectance, defined as the ratio of the sum of interactions between species in the network (the sum of connections) to the total number of potential interactions (the number of connections) [21]. Spearman correlations and network parameters were calculated using the R packages “psych” and “igraph” [22], respectively.

**M3.5 Bacterial community composition and the core OTUs for each habitat**

We explored the bacterial community composition at the phylum level across the nine habitats. The dominant bacterial phyla (defined as the top 10 most common by relative abundance in at least one habitat) were identified for each habitat. Wilcoxon tests were performed to assess significant differences in the relative abundance of these phyla across pairwise habitats. Similar analyses were conducted at the order and genus taxonomic levels to capture finer-resolution patterns within the community.

We identified the most common and ubiquitous OTUs as the core OTUs for each habitat, based on two criteria [23]. First, we identified the 10% most abundant OTUs by total read count across all samples in each habitat. Second, only OTUs present in >55% of samples were retained. Subsequently, we calculated the proportion of the core OTUs to the total OTUs in each habitat and the relative abundance of these OTUs. To better understand potential interconnectivity among habitats, we analyzed the overlap of core bacterial OTUs across habitats and visualized the results with an UpSet plot generated using the R package “UpSetR” [15]. The phylogenetic tree of core OTUs was constructed using an approximately maximum-likelihood method with the MAFFT [24] and FastTree [25] plugins in Quantitative Insights Into Microbial Ecology (Qiime2 version 2022.2.0). The phylogenetic affiliations of the core OTUs were visualized in the circle trees, and the outer heatmap was performed using the R package “ggtree” [26]. The outermost rings were added by the R package “ggtreeExtra” [27]. For each habitat, the affiliation of core OTUs at the phylum and class levels was visualized using pie charts. Additionally, the relationships between bacterial richness or LCBD and the number of core OTUs were examined using simple linear regression.

**M3.6 The impact of climate factors on bacterial communities**

Linear mixed-effects models (LMMs) were used to assess the effects of AMT, AMP, and AMS on bacterial richness (Richness) and community uniqueness (LCBD). In the LMMs, AMT, AMP, and AMS and their interactions were considered fixed effects, while habitat type was termed a random intercept effect (y ~ AMT × AMP × AMS + (1 | habitat type)). We also performed multiple linear regression (MLR) analyses for each ecosystem to examine the key climate drivers of bacterial richness and community uniqueness. Prior to these analyses, all environmental variables were z-score standardized (i.e., mean = 0, SD = 1) for each habitat, as this standardization can make the importance of all environmental variables comparable. For the MLR analyses, the standardized regression coefficients for each variable, that is, changes in bacterial richness and community uniqueness with a 1-SD increase in AMT, AMP, AMS, and their interactions, were used to compare the effects of each variable on bacterial richness and community uniqueness.

**M3.7 Statistical analyses**

The map of sampling sites was drawn using ArcGIS Pro 3.0.2. All statistical analyses were performed using R (version 4.2.1). Wilcoxon tests were performed using “EasyStat” (https://github.com/taowenmicro/EasyStat). Bacterial alpha diversity indices, including richness, evenness, Shannon-Wiener diversity, Simpson index, and Chao1, were calculated using the “vegan” package. Beta diversity based on Bray-Curtis dissimilarity was calculated for samples within each habitat using the “vegan” package. NMDS, PERMANOVA analyses, Mantel test, and simple linear regression were performed using the R package “vegan”. Local contributions to beta diversity (LCBD) were calculated using the “adespatial” package [28]. Linear mixed-effects models were conducted using the R package “lme4” [29], and hierarchical partitioning based on these models was performed using the “glam.hp” package [30]. The R package “stats” was used to fit a multiple linear regression model. All figures were created using the R package “ggplot2” [31], except for those where the visualization method has already been specified.

**Supplementary Results**

**R1** **Conceptual model of multi-sphere interconnections**

At the cross-ecosystem scale, 10.4% of all OTUs were shared between the lakes and atmosphere (Fig. 2A), which accounted for 90.2% and 64.3% of the total OTU abundance detected in the atmosphere and lakes, respectively (Fig. S3A). OTUs shared by the lakes and atmosphere and detected in the soils exhibited a mean relative abundance of 61.7% in the lakes and 65.5% in the soils (Fig. S3A). Similarly, OTUs shared by the lakes and atmosphere and occurring in the glaciers showed a mean relative abundance of 59.8% in the lakes and 87.5% in the glaciers. For OTUs shared by the lakes and atmosphere and also detected in the rivers, the mean relative abundance was 61.8% in the lakes and 77.9% in the rivers.

At the cross-ecosystem scale, 10.6% of all OTUs were shared between the soils and atmosphere (Fig. 2A), which accounted for 87.6% and 69.2% of the total OTU abundance detected in the atmosphere and soils, respectively (Fig. S3B). OTUs shared by the soils and atmosphere and also detected in the glaciers exhibited a mean relative abundance of 63.8% in the soils and 84.9% in the glaciers (Fig. S3B). Similarly, OTUs shared between the soils and atmosphere and occurring in the lakes showed a mean relative abundance of 65.5% in the soils and 61.7% in the lakes. For OTUs shared by the soils and atmosphere and also detected in the rivers, the mean relative abundance was 66.5% in the soils and 74.2% in the rivers.

At the cross-ecosystem scale, 11.6% of all OTUs were shared between the rivers and atmosphere (Fig. 2A), which accounted for 92.2% and 78.7% of the total OTU abundance detected in the atmosphere and rivers, respectively (Fig. S3C). OTUs shared by the rivers and atmosphere, and also detected in the soils, exhibited a mean relative abundance of 74.2% in the rivers and 66.5% in the soils (Fig. S3C). Similarly, OTUs shared by the rivers and atmosphere, and also occurring in the lakes, showed a mean relative abundance of 77.9% in the rivers and 61.8% in the lakes. For OTUs shared by rivers and the atmosphere and also detected in glaciers, the mean relative abundance was 75.8% in rivers and 87.7% in glaciers.

We calculated two quantitative metrics for each path under the four scenarios one by one, and the results showed: Metric 1: When glaciers serve as the intermediate system, the total relative abundance of OTUs shared between glaciers and the atmosphere is highest in the atmosphere (93.5%), followed by rivers (92.2%), lakes (90.2%), and soils (87.6%) as the intermediate system. Similarly, when glaciers serve as the intermediate system, the total relative abundance of OTUs shared between glaciers and the atmosphere is highest in glaciers (89.5%), followed by rivers (78.7%), soils (69.2%), and lakes (64.3%). Metric 2: Using any one of the habitats—lakes, soils, rivers, or glaciers—as the intermediate system, the sum of the total relative abundances in the intermediate system of atmosphere-derived OTUs shared between the intermediate system and the three "downstream systems" was calculated. The results show that when glaciers are used as the intermediate system, the sum of the three paths is the highest (260.1%), followed by rivers (227.9%), while lakes (183.3%) and soil (195.8%) are the lowest. In summary, the path from the atmosphere to glaciers is relatively ideal, and the scenario in which glaciers serve as the intermediate system also has the greatest relative potential (Fig. S3D).

Additionally, we analyzed the potential for glaciers to influence lake microbial composition via rivers. Based on the presence/absence data of OTUs, the percentage of indirectly transported OTUs (46.4%) relative to the total number of glacier OTUs is significantly higher than that of directly transported OTUs (10.2%, Fig. S4A). Based on OTU abundance data, the numbers of OTUs from the two sources differ substantially, whereas their contributions to the total OTU abundances in the lakes differ even more markedly (Fig. S4B). Indirectly transported OTUs exhibited the highest relative abundance in lakes, reaching 67.7%, whereas directly transported OTUs showed the lowest relative abundance in lakes, at only 3.9% (Fig. S4B). We hypothesize that indirectly transported OTUs, having undergone filtering in rivers, may exhibit enhanced adaptability to lake environments. Additionally, we observed that non-glacial OTUs present in rivers can be transported to lakes; their relative abundance in lakes (14.1%) is comparable to that in rivers (12.4%, Fig. S4B). The relative abundances of these OTUs remain stable in both rivers and downstream lakes, suggesting that their adaptation to riverine environments may help maintain their fitness in lakes.

**R2 Dominant bacterial orders**

The relative abundance of the dominant bacterial orders (i.e., the top 10% most abundant across all habitats) generally differed significantly among the nine habitats (Fig. S5). For example, Burkholderiales was most abundant in glacial runoff (28.6%), followed by ice-snow (24.1%), cryoconite (23.9%), and river water (23.1%), and finally lake water (14.1%), soil (11.3%), river sediment (11.2%), aerosol (9.3%) and lake sediment (8.3%). Flavobacteriales had the highest relative abundance in the glacial runoff (17.9%), river water (16.1%), and cryoconite (10.7%), followed by ice-snow (9.4%), lake water (7.2%), and river sediment (6.2%), and then aerosol (3.0%), soil (2.0%) and lake sediment (1.2%). Sphingomonadales had the highest relative abundance in ice-snow (13.3%), glacial runoff (11.1%), and river sediment (10.4%). In other habitats, its abundance was lower than 10%. Pseudomonadales’ relative abundance peaked in ice-snow (17.2%), and that in other habitats was lower than 10%. The relative abundance of Micrococcales in lake water was the highest (9.8%), followed by river sediment (7.3%) and aerosol (6.2%). In other habitats, it was lower than 5%. Cytophagales had the lowest relative abundance in lake sediment (0.9%), and Chitinophagales had the lowest relative abundance in ice-snow (0.9%). The relative abundance of Rhizobiales was lowest in cryoconite (0.3%).

**R3 Dominant bacterial genus**

The relative abundance of the dominant bacterial genus (i.e., the top 10% most abundant across all habitats) differed significantly among the nine habitats (Fig. S6). For example, *Flavobacterium* was most abundant in glacial runoff (17.6%), followed by river water (14.7%) and cryoconite (10.5%), whereas in other habitats it did not exceed 10%. The relative abundance of *Polaromonas* in glacial runoff was the highest (11.8%), followed by cryoconite (9.1%) and ice-snow (9.0%). The abundance in other habitats was lower than 5%. *Rhodoferax* had the highest relative abundance in glacial runoff (7.7%) and river water (6.5%), whereas in other habitats it was below 5%. *Acinetobacter*’s relative abundance peaked in ice-snow (12.1%) and reached the lowest level in soil (0.06%). The relative abundance of *Sphingorhabdus* in each habitat did not exceed 5%. The relative abundance of *Sphingomonas* was highest in ice-snow and lower than 5% in other habitats. The relative abundance of *Brevundimonas* was lowest in soil (0.2%) and lake sediment (0.2%).

**R4 The top 10% most abundant phyla**

The relative abundance of the top 10% most abundant phyla varied markedly among the nine habitats (Fig. 3 and Fig. S7). Proteobacteria was most abundant in ice-snow (63.9%) and glacial runoff (55.4%), followed by river water (41.4%), lake water (40.7%), cryoconite (40.6%), river sediment (39.1%), aerosol (38.0%), soil (28.1%), and lake sediment (25.8%). Bacteroidota had the highest relative abundance in the glacial runoff (28.7%,) river water (27.9%), cryoconite (27.7%), followed by ice-snow (18.8%), then lake water (13.7%), river sediment (13.5%), soil (12.6%), and lake sediment (12.0%), with the lowest abundance in aerosol (9.0%). Actinobacteriota were most abundant in lake water (22.7%), followed by aerosol (16.7%), river sediment (16.0%), soil (14.5%), and lake sediment (11.3%), whereas their abundance in other habitats was below 10.0%. Cyanobacteria peaked in cryoconite (18.6%), followed by lake water (8.6%), aerosol (8.3%), and ice-snow (5.3%), and did not exceed 5% in other habitats. Firmicutes were most abundant in aerosol (17.1%), far exceeding their abundance in other habitats (<10.0%). The relative abundance of Acidobacteriota was highest in soil (14.3%), at least twofold higher than in other habitats (<7.0%). The relative abundance of Chloroflexi was 9.4% in lake sediment and 7.3% in soil, and less than 5.0% in other habitats. Desulfobacterota had the highest relative abundance in lake sediment (5.3%), significantly exceeding that in other habitats (<1.5%).

**R5 The topology parameters of bacterial co-occurrence networks**

In bacterial co-occurrence networks, the number of effective species (network node count) was highest in lake sediment (8263±278) and soil (8263±499), significantly exceeding those in other habitats (Fig. S11A). The co-occurrence networks of ice-snow had the highest network complexity (connectance), followed by soil, river sediment, cryoconite, glacial runoff, river water, lake water, aerosol, and lake sediment (Fig. S11B). We found that the communities exhibit a pronounced trade-off between species number and effective connectance (Fig. S11C).

**R6 Taxonomic affiliations of core OTUs**

The taxonomic affiliations of core OTUs varied considerably among habitats (Fig. S12). At the phylum level, Proteobacteria is the predominant OTU taxon in all habitats, accounting for more than half of the core OTUs in ice-snow (71.9%), lake water (69.6%), glacial runoff (63.5%), and cryoconite (53.1%). At the class level, Gammaproteobacteria were the most prevalent core lineage in most habitats, except in aerosol and river sediment (Fig. S13).

Different habitats exhibited characteristic core phyla. In glacial habitats, Bacteroidota is the second most common core phylum after Proteobacteria, accounting for 29.2%, 24.2%, and 14.6% of cryoconite, glacial runoff, and ice-snow, respectively. Bacteroidota is likewise the second-most-common core phylum in river water. Actinobacteria ranked second in aerosol (34.3%), lake sediment (28.6%), soil (25.3%), lake water (21.7%), and river sediment (16.6%). Acidobacteriota was the dominant phylum in soil and river sediment, accounting for 20.0% and 10.8% of the core OTUs, respectively. Chloroflexi constituted 21.4% of the core OTUs in lake sediment, while Firmicutes accounted for 11.8% and 14.3% of the core OTUs in aerosol and lake sediment, respectively.

**R7 Phylogenetic information of Core OTUs in each habitat**

Cryoconite and glacial runoff shared the most core OTUs (shared core OTUs number: 104), followed by river water and river sediment (shared core OTUs number: 65) (Fig. S12). In these 104 core OTUs shared by cryoconite and glacial runoff, 53 OTUs belonged to Gammaproteobacteria, 29 OTUs belonged to Bacteroidia, 13 OTUs belonged to Alphaproteobacteria, and 9 OTUs belonged to other classes. At the genus level, 14 OTUs belonged to *Ferruginibacter* and 32 OTUs to the genus *Polaromonas.* In those 65 core OTUs shared by river water and river sediment, 22 OTUs belonged to Alphaproteobacteria, 9 OTUs belonged to Planctomycetes, 8 OTUs belonged to Verrucomicrobiae, and 26 OTUs belonged to other classes. At the genus level, 7 OTUs belonged to the genus *Sphingorhabdus*, 4 OTUs to the genus Novosphingobium, and 4 OTUs to the genus *Luteolibacter.*

The most shared core OTUs among the three habitats were cryoconite, glacial runoff, and river water (shared core OTUs number: 37), followed by river water, river sediment, and glacial runoff (shared core OTUs number: 24), and then ice-snow, cryoconite, and glacial runoff (shared core OTUs number: 21) (Fig. S12). In these 37 core OTUs shared by cryoconite, glacial runoff, and river water, 8 OTUs belonged to *Flavobacterium*, 6 OTUs belonged to the genus *Rhodoferax*, and 4 OTUs belonged to the genus *Pedobacter.* At the class level, 18 OTUs belonged to Gammaproteobacteria, and 15 OTUs belonged to Bacteroidia. In these 24 core OTUs shared by river water, river sediment, and glacial runoff, 10 OTUs belonged to Alphaproteobacteria, and 6 OTUs belonged to Gammaproteobacteria*.* In these 21 core OTUs shared by ice-snow, cryoconite, and glacial runoff, 6 OTUs belonged to the genus *Polaromonas*, 4 OTUs belonged to the genus *Hymenobacter*, and 3 OTUs belonged to the genus *Massilia.* At the class level, 11 OTUs belonged to Gammaproteobacteria.

The most shared core OTUs among the four habitats were cryoconite, glacial runoff, river water, and river sediment (shared core OTUs number: 34), followed by ice-snow, cryoconite, glacial runoff, and river water (shared core OTUs number: 7) (Fig. S12). In these 34 core OTUs shared by cryoconite, glacial runoff, river water, and river sediment, 14 OTUs belonged to Bacteroidia, 9 OTUs belonged to Alphaproteobacteria, and 6 OTUs belonged to Gammaproteobacteria. At the genus level, 7 OTUs belonged to *Flavobacterium*, 5 OTUs belonged to *Sphingorhabdus*, and 5 OTUs belonged to *Ferruginibacter.* In these seven core OTUs shared by ice-snow, cryoconite, glacial runoff, and river water, 2 OTUs belonged to *Polaromonas,* and 6 OTUs belonged to Gammaproteobacteria.

**R8 The microbial communities respond to the climate factors of each ecosystem**

**R8.1 The single and joint effects of climate factors on bacterial species richness**

The effects of AMT on bacterial richness were significantly positive in glaciers but significantly negative in rivers, lakes, and soil (Fig. S15). The effects of AMT on bacterial richness were not significant in the atmosphere. Bacterial richness in soil and lake had a stronger response to AMT changes. Precisely, when AMT increased one standard deviation (SD), bacterial richness in soil and lake decreased by 0.5 and 0.4 SD (soil: β = -0.5, *P* < 0.001; lake: β = -0.4, *P* < 0.001), respectively, while bacterial richness in other ecosystems changed by less than 0.3 SD (|β| < 0.3).

The effects of AMS on bacterial richness were significantly positive in the atmosphere but negative in the glaciers (Fig. S15). The effects of AMS on bacterial richness were not statistically significant in other ecosystems. Bacterial richness in the atmosphere had a stronger response to AMS changes. Precisely, when AMS increased one standard deviation (SD), bacterial richness in the atmosphere increased 0.3 SD (atmosphere: β = 0.3, *P* = 0.03), while bacterial richness in other ecosystems changed and did not exceed 0.3 SD (|β| < 0.3).

The joint effects of AMT and AMP on bacterial richness were significantly negative in glaciers but not significant in other ecosystems (Fig. S15). Bacterial richness in glaciers exhibited a stronger response to combined changes in AMT and AMP. Precisely, when joint AMT and AMP increased by one standard deviation (SD), bacterial richness in glaciers decreased by 0.3 SD (β = -0.3, *P* < 0.001), while bacterial richness in other ecosystems changed by less than 0.2 SD (|β| < 0.2).

The joint effects of AMT and AMS on bacterial richness were significantly negative in glaciers and lakes but not significant in other ecosystems (Fig. S15). Bacterial richness in glaciers and lakes responded more strongly to combined changes in AMT and AMS. Precisely, when joint AMT and AMS increased one standard deviation (SD), bacterial richness in glaciers and lakes decreased by 0.2 SD (β = -0.2, *P* < 0.01), while bacterial richness in other ecosystems changed by less than 0.12 SD (|β| < 0.12).

The joint effects of AMP and AMS on bacterial richness were significantly positive in glaciers and rivers, while negative in soil (Fig. S15). The joint effects of AMP and AMS on bacterial richness were non-significant in other ecosystems. Bacterial richness in glaciers showed a stronger response to joint changes in AMP and AMS. Precisely, when joint AMP and AMS increased one standard deviation (SD), bacterial richness in the glacier increased 0.5 SD (β = 0.5, *P* < 0.001), while bacterial richness in other ecosystems changed by less than 0.45 SD (|β| < 0.45).

The joint effects of AMT, AMP, and AMS on bacterial richness were significantly positive in soil but negative in the river (Fig. S15). The joint effects of AMT, AMP, and AMS on bacterial richness were non-significant in other ecosystems. Bacterial richness in the river exhibited a stronger response to combined changes in AMT, AMP, and AMS. Specifically, when joint AMT, AMP, and AMS increased by one standard deviation (SD), bacterial richness in the river decreased by 0.15 SD (β = -0.15, *P* = 0.005), while bacterial richness in other ecosystems changed by less than 0.13 SD (|β| < 0.13).

**R8.2 The single and joint effects of climate factors on LCBD**

Effects of AMT on LCBD were significantly positive in the atmosphere, lake, and soil, while significantly negative in the glacier (Fig. S16). Effects of AMT on LCBD were non-significant in the river. The lake's community uniqueness (LCBD) responded most vigorously to AMT changes. Specifically, when AMT increased by one standard deviation (SD), LCBD in the lake increased by 0.9 SD (β = 0.9, *P* < 0.001), while LCBD in other habitats changed by less than 0.5 SD (|β| < 0.5).

The effects of AMS on LCBD were significantly positive in the glacier ecosystem, whereas they were significantly negative in other ecosystems (Fig. S16). Community uniqueness (LCBD) in glaciers and the atmosphere responded strongly to changes in AMS. Specifically, when AMS increased by one standard deviation (SD), LCBD in glacier and atmosphere changed by 0.5 and 0.6 SD (glacier: β = 0.5, *P* = 0.003; atmosphere: β = -0.6, *P* < 0.001), while LCBD in other habitats changed by less than 0.25 SD (|β| < 0.25).

The joint effects of AMT and AMP on LCBD were significantly positive in the glacier, river, and soil, whereas they were significantly negative in the atmosphere (Fig. S16). The joint effects of AMT and AMP on LCBD were non-significant in the lake. The atmosphere's community uniqueness (LCBD) responded strongly to joint changes in AMT and AMP. Specifically, when joint AMT and AMP increased by one standard deviation (SD), LCBD in the atmosphere decreased by 0.9 SD (β = -0.9, *P* = 0.02), while LCBD in other habitats changed by less than 0.5 SD (|β| < 0.5).

The joint effects of AMT and AMS on LCBD were significantly positive in the river, lake, and soil (Fig. S16). The joint effects of AMT and AMS on LCBD were non-significant in other ecosystems. The soil community's uniqueness (LCBD) responded strongly to joint changes in AMT and AMS. Specifically, when joint AMT and AMS increased by one standard deviation (SD), LCBD in the soil increased by 0.3 SD (β = 0.3, *P* < 0.001), while LCBD in other habitats changed by less than 0.2 SD (|β| < 0.2).

The joint effects of AMP and AMS on LCBD were significantly positive in the lake, whereas they were significantly negative in the river and glacier (Fig. S16). The joint effects of AMP and AMS on LCBD were non-significant in other ecosystems. The community uniqueness (LCBD) of glaciers showed the strongest response to the joint changes in AMP and AMS. Specifically, when joint AMP and AMS increased one standard deviation (SD), LCBD in glaciers decreased by 0.8 SD (β = -0.8, *P* < 0.001), while LCBD in other habitats changed by less than 0.4 SD (|β| < 0.4).

The joint effects of AMT, AMP, and AMS on LCBD were significantly positive in the river, while significantly negative in the lake and soil (Fig. S16). Joint effects of AMT, AMP, and AMS on LCBD were non-significant in other ecosystems. The lake's community uniqueness (LCBD) responded strongly to changes in joint AMT, AMP, and AMS. Specifically, when joint AMT, AMP, and AMS increased by one standard deviation (SD), LCBD in the lake decreased by 0.3 SD (β = -0.3, *P* = 0.004), while LCBD in the river and sediment changed by less than 0.1 SD (|β| < 0.1).

**Supplementary Tables and Figures**

**Table S1 Samples collected from five ecosystems and nine habitats for construction of the catalogue.**

| Ecosystem | Habitat | Sample size (n) | Status | Database | Accession no. | Reference |
| --- | --- | --- | --- | --- | --- | --- |
| Glacier | Ice-snow | 241 | Unpublished | NCBI | PRJNA1233668 | this study |
|  | Cryoconite | 257 | Unpublished | NCBI | PRJNA1233668 | this study |
|  | Glacial runoff | 132 | Unpublished | NCBI | PRJNA1233668 | this study |
| River | River water | 60 | Published | NCBI | PRJNA608087 | [4] |
|  |  | 303 | Unpublished | NCBI | PRJNA1233668 | this study |
|  | River sediment | 138 | Published | NCBI | PRJNA790017, PRJNA608087 | [4,32] |
|  |  | 59 | Unpublished | NCBI | PRJNA1233668 | this study |
| Lake | Lake water | 42 | Published | NCBI | PRJNA1056829, PRJNA608087 | [4] |
|  |  | 673 | Unpublished | NCBI | PRJNA1233668 | this study |
|  | Lake sediment | 323 | Published | NCBI | PRJNA1056829, PRJNA608087 | [4,33] |
|  |  | 193 | Unpublished | NCBI | PRJNA1233668 | this study |
| Soil | Soil | 132 | Published | NCBI | PRJNA980427 | [34] |
|  |  | 642 | Unpublished | NCBI | PRJNA1233668 | this study |
| Atmosphere | Aerosol | 54 | Published | NCBI | PRJNA1013198 | [35] |
|  |  | 124 | Unpublished | NCBI | PRJNA1233668 | this study |

**Table S2 The taxonomy of the shared core OTUs.**

| OTU ID | Phylum | Class | Order | Family | Genus | Species |
| --- | --- | --- | --- | --- | --- | --- |
| OTU4 | Proteobacteria | Gammaproteobacteria | Burkholderiales | Comamonadaceae | *Polaromonas* | - |
| OTU52 | Actinobacteriota | Actinobacteria | Micrococcales | Microbacteriaceae | *Cryobacterium* | *Cryobacterium psychrotolerans* |
| OTU7 | Proteobacteria | Gammaproteobacteria | Burkholderiales | Comamonadaceae | *Rhodoferax* | - |
| OTU8 | Actinobacteriota | Actinobacteria | Micrococcales | Micrococcaceae | *Pseudarthrobacter* | - |

**Table S3 Results of PERMANOVA analyses, showing the differences in community composition among the eleven habitats.**

| group1 | group2 | Df | Sums of squares | Mean squares | F.Model | Variation (R^2^) | Pr (>F) | P_adj_BH |
| --- | --- | --- | --- | --- | --- | --- | --- | --- |
| Aerosol | River water | 1 | 21.2 | 21.2 | 57.5 | 0.096 | 0.001 | 0.001 |
| Aerosol | River sediment | 1 | 11.6 | 11.6 | 29.8 | 0.074 | 0.001 | 0.001 |
| Aerosol | Lake water | 1 | 18.1 | 18.1 | 41.4 | 0.044 | 0.001 | 0.001 |
| Aerosol | Lake sediment | 1 | 14.8 | 14.8 | 33.5 | 0.046 | 0.001 | 0.001 |
| Aerosol | Soil | 1 | 16.4 | 16.4 | 38.1 | 0.039 | 0.001 | 0.001 |
| Aerosol | Cryoconite | 1 | 19.7 | 19.7 | 54.0 | 0.111 | 0.001 | 0.001 |
| Aerosol | Ice-snow | 1 | 12.1 | 12.1 | 30.8 | 0.069 | 0.001 | 0.001 |
| Aerosol | Glacial runoff | 1 | 16.0 | 16.0 | 46.0 | 0.13 | 0.001 | 0.001 |
| River water | River sediment | 1 | 16.7 | 16.7 | 45.4 | 0.075 | 0.001 | 0.001 |
| River water | Lake water | 1 | 27.2 | 27.2 | 65.0 | 0.057 | 0.001 | 0.001 |
| River water | Lake sediment | 1 | 30.4 | 30.4 | 72.9 | 0.077 | 0.001 | 0.001 |
| River water | Soil | 1 | 37.5 | 37.5 | 90.9 | 0.074 | 0.001 | 0.001 |
| River water | Cryoconite | 1 | 26.3 | 26.3 | 74.7 | 0.108 | 0.001 | 0.001 |
| River water | Ice-snow | 1 | 22.7 | 22.7 | 61.2 | 0.092 | 0.001 | 0.001 |
| River water | Glacial runoff | 1 | 15.1 | 15.1 | 44.7 | 0.083 | 0.001 | 0.001 |
| River sediment | Lake water | 1 | 19.0 | 19.0 | 43.7 | 0.046 | 0.001 | 0.001 |
| River sediment | Lake sediment | 1 | 12.1 | 12.1 | 27.5 | 0.037 | 0.001 | 0.001 |
| River sediment | Soil | 1 | 13.1 | 13.1 | 30.7 | 0.031 | 0.001 | 0.001 |
| River sediment | Cryoconite | 1 | 19.9 | 19.9 | 54.8 | 0.108 | 0.001 | 0.001 |
| River sediment | Ice-snow | 1 | 16.4 | 16.4 | 42.1 | 0.088 | 0.001 | 0.001 |
| River sediment | Glacial runoff | 1 | 14.2 | 14.2 | 40.9 | 0.111 | 0.001 | 0.001 |
| Lake water | Lake sediment | 1 | 21.3 | 21.3 | 47.1 | 0.037 | 0.001 | 0.001 |
| Lake water | Soil | 1 | 34.1 | 34.1 | 77.0 | 0.049 | 0.001 | 0.001 |
| Lake water | Cryoconite | 1 | 29.8 | 29.8 | 70.8 | 0.068 | 0.001 | 0.001 |
| Lake water | Ice-snow | 1 | 20.3 | 20.3 | 46.8 | 0.047 | 0.001 | 0.001 |
| Lake water | Glacial runoff | 1 | 20.8 | 20.8 | 49.1 | 0.055 | 0.001 | 0.001 |
| Lake sediment | Soil | 1 | 15.0 | 15.0 | 33.6 | 0.025 | 0.001 | 0.001 |
| Lake sediment | Cryoconite | 1 | 27.5 | 27.5 | 65.3 | 0.078 | 0.001 | 0.001 |
| Lake sediment | Ice-snow | 1 | 19.9 | 19.9 | 45.6 | 0.057 | 0.001 | 0.001 |
| Lake sediment | Glacial runoff | 1 | 21.1 | 21.1 | 49.7 | 0.071 | 0.001 | 0.001 |
| Soil | Cryoconite | 1 | 35.0 | 35.0 | 84.3 | 0.076 | 0.001 | 0.001 |
| Soil | Ice-snow | 1 | 24.8 | 24.8 | 58.0 | 0.054 | 0.001 | 0.001 |
| Soil | Glacial runoff | 1 | 24.0 | 24.0 | 57.6 | 0.06 | 0.001 | 0.001 |
| Cryoconite | Ice-snow | 1 | 15.0 | 15.0 | 40.7 | 0.076 | 0.001 | 0.001 |
| Cryoconite | Glacial runoff | 1 | 9.3 | 9.3 | 28.4 | 0.068 | 0.001 | 0.001 |
| Ice-snow | Glacial runoff | 1 | 8.8 | 8.8 | 24.8 | 0.063 | 0.001 | 0.001 |

**Table S4 Summary of hierarchical partitioning based on linear mixed models.**

| Response variables | Influencing factors | Unique | Average shared | Individual | I.perc(%) | P-value |
| --- | --- | --- | --- | --- | --- | --- |
| Richness | AMT | 0.0031 | -0.0001 | 0.003 | 3.07 | ＜0.001 |
|  | AMP | 0.0646 | 0.0007 | 0.0653 | 66.84 | ＜0.001 |
|  | AMS | 0.0032 | 0.0057 | 0.0089 | 9.11 | 0.001 |
|  | AMT×AMP | 0.0051 | 0.0015 | 0.0066 | 6.76 | ＜0.001 |
|  | AMT×AMS | 0.0025 | 0.0016 | 0.0041 | 4.20 | ＜0.001 |
|  | AMP×AMS | 0.0052 | 0.0013 | 0.0065 | 6.65 | ＜0.001 |
|  | AMT×AMP×AMS | 0.0000 | 0.0033 | 0.0033 | 3.38 | ns |
| LCBD | AMT | 0.0347 | -0.0123 | 0.0224 | 13.96 | ＜0.001 |
|  | AMP | 0.1230 | -0.0353 | 0.0877 | 54.64 | ＜0.001 |
|  | AMS | 0.0312 | -0.0177 | 0.0135 | 8.41 | ＜0.001 |
|  | AMT×AMP | 0.0171 | -0.0042 | 0.0129 | 8.04 | ＜0.001 |
|  | AMT×AMS | 0.0131 | -0.0038 | 0.0093 | 5.79 | ＜0.001 |
|  | AMP×AMS | 0.0050 | 0.0019 | 0.0069 | 4.30 | ＜0.001 |
|  | AMT×AMP×AMS | 0.0001 | 0.0077 | 0.0078 | 4.86 | ns |

**Fig. S1** Upset plot for OTUs of 5 habitat types. Horizontal bars on the left represent the total number of OTUs in each habitat. The bars on the top represent the number of OTUs at each intersection, as the connected dots indicate the habitats involved.

**Fig. S2** Interactions within ecosystems of (A) glaciers, (B) lakes, and (C) rivers. The numbers along the line represent the percentage of overlapping OTUs between pairwise habitats relative to the total number of OTUs in the ecosystem. Shared OTUs were those found in all habitats of the corresponding ecosystems.

**Fig. S3** Conceptual models of multi-sphere interconnection with the (A) lake, (B) soil, (C) river, and (D) glacier ecosystems serving respectively as intermediate systems in multi-sphere interconnections.

**Fig. S4** Using an atmosphere-glacier-downstream system model to analyze the potential for glaciers to influence lake microbial composition via rivers. Left panel: presence/absence of OTUs; right panel: OTU abundances.

**Fig. S5** Relative abundances of the dominant bacterial orders (ranked among the top 10 most common orders by relative abundance in one or more habitats) for the nine habitat types.

**Fig. S6** Relative abundances of the dominant bacterial genera (with an average relative abundance ≥ 1% across all samples) for the nine habitat types.

**Fig. S7** Differences in the relative abundance of the dominant bacterial phyla among the nine habitat types. Each box's top and bottom boundaries indicate the 25^th^ and 75^th^ quartile values, respectively. Lines within the boxes represent the median values, and white-filled dots represent the mean values. Different letters indicate significant differences at the *P* < 0.05 level determined by nonparametric Wilcoxon tests.

**Fig. S8** Differences in the bacterial alpha diversity among the nine habitat types. Different letters indicate significant differences at the *P* < 0.05 level determined by nonparametric Wilcoxon tests. Each box's top and bottom boundaries indicate the 25^th^ and 75^th^ quartile values, respectively. Lines within the boxes represent the median values, and white-filled dots represent the mean values.

**Fig. S9** Relationships between local contribution to beta diversity (LCBD) and bacterial richness for each habitat. Linear or quadratic models were applied to analyze the relationships between LCBD and bacterial richness, and the most appropriate models were selected based on AIC. Statistically significant relationships are shown with solid lines (*P* < 0.05), while dashed lines show non-significant relationships (*P* ≥ 0.05).

**Fig. S10** Differences in the bacterial diversity among the nine habitat types. To avoid potential biases introduced by differences in sample sizes across habitats, we used a resampling method. For details, please refer to the Materials and Methods section of the main text. Different letters indicate significant differences at the *P* < 0.05 level determined by nonparametric Wilcoxon tests. Each box's top and bottom boundaries indicate the 25^th^ and 75^th^ quartile values, respectively. Lines within the boxes represent the median values, and white-filled dots represent the mean values.

**Fig. S11** The bacterial network topology parameters differ among the nine habitat types. Different letters indicate significant differences at the *P* < 0.05 level determined by nonparametric Wilcoxon tests. Each box's top and bottom boundaries indicate the 25^th^ and 75^th^ quartile values, respectively. Lines within the boxes represent the median values, and white-filled dots represent the mean values.

**Fig. S12** Upset plot for core OTUs of 9 habitat types. Horizontal bars on the left represent the total number of core OTUs in each habitat. The bars on the top represent the number of core OTUs of each intersection, as indicated by the connected dots.

**Fig. S13** The taxonomic affiliations of the core OTUs (A) at the phylum level and (B) at the class level.

**Fig. S14** Percentage (number of OTUs) and relative abundance (reads of OTUs) of the core OTUs versus the total microbial OTUs under nine habitat types. The number of core OTUs in the corresponding habitat is shown in the bottom-right corner.

**Fig. S15** Effects of climatic variables on bacterial richness based on multiple linear regression for each ecosystem. The panel shows coefficients (dots) and 95% CIs (bars) from multiple linear regressions, illustrating the standardized effects of AMT, AMP, and AMS, and their interaction, on bacterial richness. All predictors were standardized before conducting the multiple linear regression. T×P: the interaction of AMT and AMP; T×S: the interaction of AMT and AMS; P×S: the interaction of AMP and AMS; T×P×S: the interaction of AMT, AMP, and AMS.

**Fig. S16** Effects of climatic variables on bacterial LCBD based on multiple linear regression for each ecosystem. The panel shows the coefficients (dots) and 95% CIs (bars) from multiple linear regressions, illustrating the standardized effects of AMT, AMP, and AMS, and their interaction, on LCBD. All predictors were standardized before conducting the multiple linear regression. T×P: the interaction of AMT and AMP; T×S: the interaction of AMT and AMS; P×S: the interaction of AMP and AMS; T×P×S: the interaction of AMT, AMP, and AMS.

**Fig. S17** Relationships between the Bray-Curtis dissimilarity and geographic distance by (A) Mantel tests and (B) Partial Mantel analyses for each habitat. Statistically significant values are marked by asterisks: ****P* < 0.001, **0.001 ≤ *P*  < 0.01, *0.01 ≤ *P*  <  0.05.

**Reference**

1.	Liu KS, Liu YQ and Hu AY *et al*. Fate of glacier surface snow-originating bacteria in the glacier-fed hydrologic continuums. *Environ Microbiol* 2021; 23: 6450-62. doi: 10.1111/1462-2920.15788

2.	Liao X, Hou LY and Zhang LP *et al*. Distinct influences of altitude on microbiome and antibiotic resistome assembly in a glacial river ecosystem of Mount Everest. *J Hazard Mater* 2024; 479: 135675. doi: 10.1016/j.jhazmat.2024.135675

3.	Qi J, Huang ZW and Maki T *et al*. Airborne bacterial communities over the Tibetan and Mongolian Plateaus: variations and their possible sources. *Atmos Res* 2021; 247: 105215. doi: 10.1016/j.atmosres.2020.105215

4.	Zhang LY, Delgado-Baquerizo M and Shi Y *et al*. Co-existing water and sediment bacteria are driven by contrasting environmental factors across glacier-fed aquatic systems. *Water Res* 2021; 198: 117139. doi: 10.1016/j.watres.2021.117139

5.	Liu X, Zhang CZ and Yang T *et al*. Phylogenetic relatedness enhances the understanding of soil microbial coexistence in alpine wetlands of the Tibetan Plateau. *Soil Biol Biochem* 2023; 185: 109160. doi: 10.1016/j.soilbio.2023.109160

6.	Liu YQ, Ji MK and Yu T *et al*. A genome and gene catalog of glacier microbiomes. *Nat Biotechnol* 2022; 40: 1341-8. doi: 10.1038/s41587-022-01367-2

7.	Bolger AM, Lohse M and Usadel B. Trimmomatic: a flexible trimmer for Illumina sequence data. *J Bioinform* 2014; 30: 2114-20. doi: 10.1093/bioinformatics/btu170

8.	Magoč T and Salzberg SL. FLASH: fast length adjustment of short reads to improve genome assemblies. *J Bioinform* 2011; 27: 2957-63. doi: 10.1093/bioinformatics/btr507

9.	Martin M. Cutadapt removes adapter sequences from high-throughput sequencing reads. *EMBnet J* 2011; 17: 10-2. doi: 10.14806/ej.17.1.200

10.	Deng YC, Umbach AK and Neufeld JD. Nonparametric richness estimators Chao1 and ACE must not be used with amplicon sequence variant data. *ISME J* 2024; 18: wrae106. doi: 10.1093/ismejo/wrae106

11.	Rognes T, Flouri T and Nichols B *et al*. VSEARCH: a versatile open source tool for metagenomics. *PeerJ* 2016; 4: e2584. doi: 10.7717/peerj.2584

12.	Edgar RC, Haas BJ and Clemente JC *et al*. UCHIME improves sensitivity and speed of chimera detection. *J Bioinform* 2011; 27: 2194-200. doi: 10.1093/bioinformatics/btr381

13.	Edgar RC. Search and clustering orders of magnitude faster than BLAST. *J Bioinform* 2010; 26: 2460-1. doi: 10.1093/bioinformatics/btq461

14.	Fick SE and Hijmans RJ. WorldClim 2: new 1‐km spatial resolution climate surfaces for global land areas. *Int J Climatol* 2017; 37: 4302-15. doi: 10.1002/joc.5086

15.	Conway JR, Lex A and Gehlenborg N. UpSetR: an R package for the visualization of intersecting sets and their properties. *J Bioinform* 2017; 33: 2938-40. doi: 10.1093/bioinformatics/btx364

16.	Anderson MJ. A new method for non-parametric multivariate analysis of variance. *Austral Ecol* 2001; 26: 32-46. doi: 10.1111/j.1442-9993.2001.01070.pp.x

17.	Hsieh TC, Ma KH and Chao A. iNEXT: an R package for rarefaction and extrapolation of species diversity (Hill numbers). *Methods Ecol Evol* 2016; 7: 1451-6. doi: 10.1111/2041-210X.12613

18.	Legendre P and De Cáceres M. Beta diversity as the variance of community data: dissimilarity coefficients and partitioning. *Ecol Lett* 2013; 16: 951-63. doi: 10.1111/ele.12141

19.	Anderson MJ, Crist TO and Chase JM *et al*. Navigating the multiple meanings of beta diversity: a roadmap for the practicing ecologist. *Ecol Lett* 2011; 14: 19-28. doi: 10.1111/j.1461-0248.2010.01552.x

20.	Yamaoka K, Nakagawa T and Uno T. Application of Akaike's information criterion (AIC) in the evaluation of linear pharmacokinetic equations. *J Pharmacokinet Biopharm* 1978; 6: 165-75. doi: 10.1007/BF01117450

21.	Yuan MM, Guo X and Wu LW *et al*. Climate warming enhances microbial network complexity and stability. *Nat Clim Change* 2021; 11: 343-8. doi: 10.1038/s41558-021-00989-9

22.	Antonov M, Csárdi G and Horvát S *et al*. igraph enables fast and robust network analysis across programming languages. *arXiv preprint arXiv:2311.10260*. 2023. doi: 10.48550/arXiv.2311.10260

23.	Delgado-Baquerizo M, Oliverio AM and Brewer TE *et al*. A global atlas of the dominant bacteria found in soil. *Science* 2018; 359: 320-5. doi: 10.1126/science.aap9516

24.	Katoh K, Misawa K and Kuma K-I *et al*. MAFFT: a novel method for rapid multiple sequence alignment based on fast Fourier transform. *Nucleic Acids Res* 2002; 30: 3059-66. doi: 10.1093/nar/gkf436

25.	Price MN, Dehal PS and Arkin AP. FastTree: computing large minimum evolution trees with profiles instead of a distance matrix. *Mol Biol Evol* 2009; 26: 1641-50. doi: 10.1093/molbev/msp077

26.	Yu GC, Smith DK and Zhu HC *et al*. GGTREE: an R package for visualization and annotation of phylogenetic trees with their covariates and other associated data. *Methods Ecol Evol* 2017; 8: 28-36. doi: 10.1111/2041-210X.12628

27.	Xu SB, Dai ZH and Guo PF *et al*. ggtreeExtra: compact visualization of richly annotated phylogenetic data. *Mol Biol Evol* 2021; 38: 4039-42. doi: 10.1093/molbev/msab166

28.	Dray S, Bauman D and Blanchet G *et al*. adespatial: Multivariate Multiscale Spatial Analysis. *R package version 0.3-23* 2023, https://CRAN.R-project.org/package=adespatial

29.	Bates D, Mächler M and Bolker BM *et al*. Fitting linear mixed-effects models using lme4. *J Stat Softw* 2015; 67: 1-48. doi: 10.18637/jss.v067.i01

30.	Lai JS, Zou Y and Zhang JL *et al*. Generalizing hierarchical and variation partitioning in multiple regression and canonical analyses using the rdacca.hp R package. *Methods Ecol Evol* 2022; 13: 782-8. doi: 10.1111/2041-210x.13800

31.	Wickham H. ggplot2-elegant graphics for data analysis. *J Stat Softw* 2017; 77: 1-3. doi: 10.18637/jss.v077.b02

32.	Liu KS, Hu AY and Hou LY *et al*. Effects of land-use patterns on the biogeography of the sediment bacteria in the Yarlung Tsangpo River. *FEMS Microbiol Ecol* 2022; 98: fiac125. doi: 10.1093/femsec/fiac125

33.	Deng YC, Liang CL and Zhu XM *et al*. Methylomonadaceae was the active and dominant methanotroph in Tibet lake sediments. *ISME Commun* 2024; 4: ycae032. doi: 10.1093/ismeco/ycae032

34.	Zeng YF, Guo X and Lei JS *et al*. Regional microbial biogeography linked to soil respiration. *Sci Total Environ* 2024; 929: 172263. doi: 10.1016/j.scitotenv.2024.172263

35.	Zhang ZH, Qi J and Liu YQ *et al*. Anthropogenic impact on airborne bacteria of the Tibetan Plateau. *Environ Int* 2024; 183: 108370. doi: 10.1016/j.envint.2023.108370
